# Berberine alleviates ETEC-induced intestinal inflammation and oxidative stress damage by optimizing intestinal microbial composition in a weaned piglet model

**DOI:** 10.3389/fimmu.2024.1460127

**Published:** 2024-09-16

**Authors:** Yue Wang, Ziting Zhang, Min Du, Xu Ji, Xiaodan Liu, Chunfang Zhao, Xunsheng Pang, Erhui Jin, Aiyou Wen, Shenghe Li, Feng Zhang

**Affiliations:** ^1^ College of Animal Science, Anhui Science and Technology University, Chuzhou, China; ^2^ Anhui Province Key Laboratory of Livestock and Poultry Product Safety Engineering, Institute of Animal Science and Veterinary Medicine, Anhui Academy of Agricultural Sciences, Hefei, China; ^3^ Anhui Province Key Laboratory of Animal Nutrition Regulation and Health, Anhui Science and Technology University, Chuzhou, China

**Keywords:** enterotoxigenic *Escherichia coli*, berberine, weaned piglet, intestinal inflammation, oxidative damage

## Abstract

**Introduction:**

Enterotoxigenic *Escherichia coli* (ETEC) is the main diarrhea-causing pathogen in children and young animals and has become a global health concern. Berberine is a type of “medicine and food homology” and has a long history of use in China, particularly in treating gastrointestinal disorders and bacterial diarrhea.

**Methods:**

In this study, we explored the effects of berberine on growth performance, intestinal inflammation, oxidative damage, and intestinal microbiota in a weaned piglet model of ETEC infection. Twenty-four piglets were randomly divided into four groups—a control group (fed a basal diet [BD] and infused with saline), a BD+ETEC group (fed a basal diet and infused with ETEC), a LB+ETEC group (fed a basal diet with 0.05% berberine and infused with ETEC infection), and a HB+ETEC group (fed a basal diet with 0.1% berberine and infused with ETEC).

**Results:**

Berberine significantly improved the final body weight (BW), average daily gain (ADG), and average daily feed intake (ADFI) (*P*<0.05) of piglets, and effectively decreased the incidence of diarrhea among the animals (*P*<0.05). Additionally, berberine significantly downregulated the expression levels of the genes encoding TNF-α, IL-1β, IL-6, IL-8, TLR4, MyD88, NF-κB, IKKα, and IKKβ in the small intestine of piglets (*P*<0.05). ETEC infection significantly upregulated the expression of genes coding for Nrf2, CAT, SOD1, GPX1, GST, NQO1, HO-1, GCLC, and GCLM in the small intestine of the animals (*P*<0.05). Berberine significantly upregulated 12 functional COG categories and 7 KEGG signaling pathways. A correlation analysis showed that berberine significantly increased the relative abundance of beneficial bacteria (Gemmiger, Pediococcus, Levilactobacillus, Clostridium, Lactiplantibacillus, Weissella, Enterococcus, Blautia, and Butyricicoccus) and decreased that of pathogenic bacteria (Prevotella, Streptococcus, Parabacteroides, Flavonifractor, Alloprevotella) known to be closely related to intestinal inflammation and oxidative stress in piglets. In conclusion, ETEC infection disrupted the intestinal microbiota in weaned piglets, upregulating the TLR4/MyD88/NF-κB and Nrf2 signaling pathways, and consequently leading to intestinal inflammation and oxidative stress-induced damage.

**Discussion:**

Our data indicated that berberine can optimize intestinal microbiota balance and modulate the TLR4/MyD88/NF-κB and Nrf2 signaling pathways, thus helping to alleviate intestinal inflammation and oxidative damage caused by ETEC infection in weaned piglets.

## Graphical Abstract

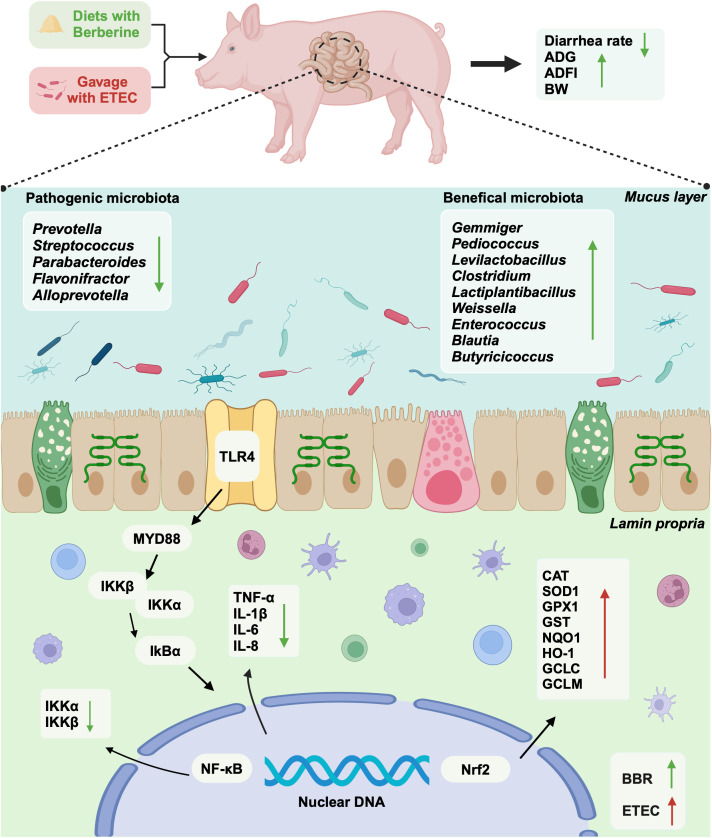

## 1 Introduction

Enterotoxigenic *Escherichia coli* (ETEC) is a recognized diarrhea-causing intestinal pathogen, particularly in children under 5 years of age, and livestock. Diarrhea due to ETEC infection presents a substantial challenge to global health (1–4). It is estimated that ETEC contributes to 220 million cases of diarrhea worldwide, with approximately 75 million occurring in children under 5 years of age, leading to an estimated 18,700 to 42,000 fatalities (2, 5). In the swine industry worldwide, ETEC is a primary factor contributing to post-weaning diarrhea in pigs, resulting in significant economic losses (6). ETEC non-invasively colonizes the cell wall of the small intestine and releases heat-stable toxins, thereby causing diarrhea (2). ETEC infection can activate Nrf2 signaling pathway and cause intestinal oxidative damage (7, 8). ETEC colonization also directly induces gut barrier dysfunction, inhibits intestinal immune function, and triggers an inflammatory response (9, 10). The management of ETEC infection through antibiotic therapy is a critical focus in microbiology and infectious disease research (11). The antibiotics used for prophylaxis against ETEC-induced diarrhea include doxycycline, ciprofloxacin, and rifaximin (5). However, given the growing concern regarding ETEC resistance to these antibiotics (3), the search for safe and effective alternatives for the prevention and treatment of ETEC infections has become an urgent public health priority.

Medicine food homology (MFH) refers to substances that possess both nutritional and medicinal attributes (12). Many secondary metabolites (e.g., alkaloids, terpenoids, polysaccharides, saponins, phenolic compounds) found in MFH plants exhibit antibacterial, anti-inflammatory, and antioxidant properties (12–15). Berberine is an alkaloid compound that has been used to treat gastrointestinal diseases, especially bacterial diarrhea, in China for hundreds of years (16, 17). Berberine has garnered attention for its effectiveness against various pathogenic microorganisms, notably Gram-negative bacteria (18–20). Its key benefits include anti-inflammatory, antioxidant, and immunomodulatory properties (20). Berberine works by inhibiting inflammatory responses through a variety of mechanisms, including reducing inflammatory cytokine production, suppressing oxidative stress, and promoting immune regulation (16, 20). To date, relatively few studies have reported on the effects of berberine in ETEC infection-induced intestinal inflammation and oxidative damage. Pigs are considered ideal models for studying a raft of human conditions given the greater similarities in organ size, anatomical structure, and physiological characteristics when compared to small experimental animals. In this study, we investigated the effects of berberine on intestinal inflammation, oxidative damage, and intestinal microbiota in weaned piglets infected with ETEC, providing potential novel strategies for the treatment of diarrhea caused by infection with this bacterium.

## 2 Materials and methods

### 2.1 Animal ethics

The animal study were approved by Experimental Animal Ethics Committee of Anhui Science and Technology University (AHSTU2023006). The study were conducted in accordance with the local legislation and institutional requirements. Written informed consent was obtained from the owners for the participation of their animals in this study.

### 2.2 Bacterial strains

The *E. coli* F4 (K88 ac) strain was purchased from the National Center for Veterinary Culture Collection of China (CVCC1500).

### 2.3 Animals, housing, and experimental design

**Figure 1:**
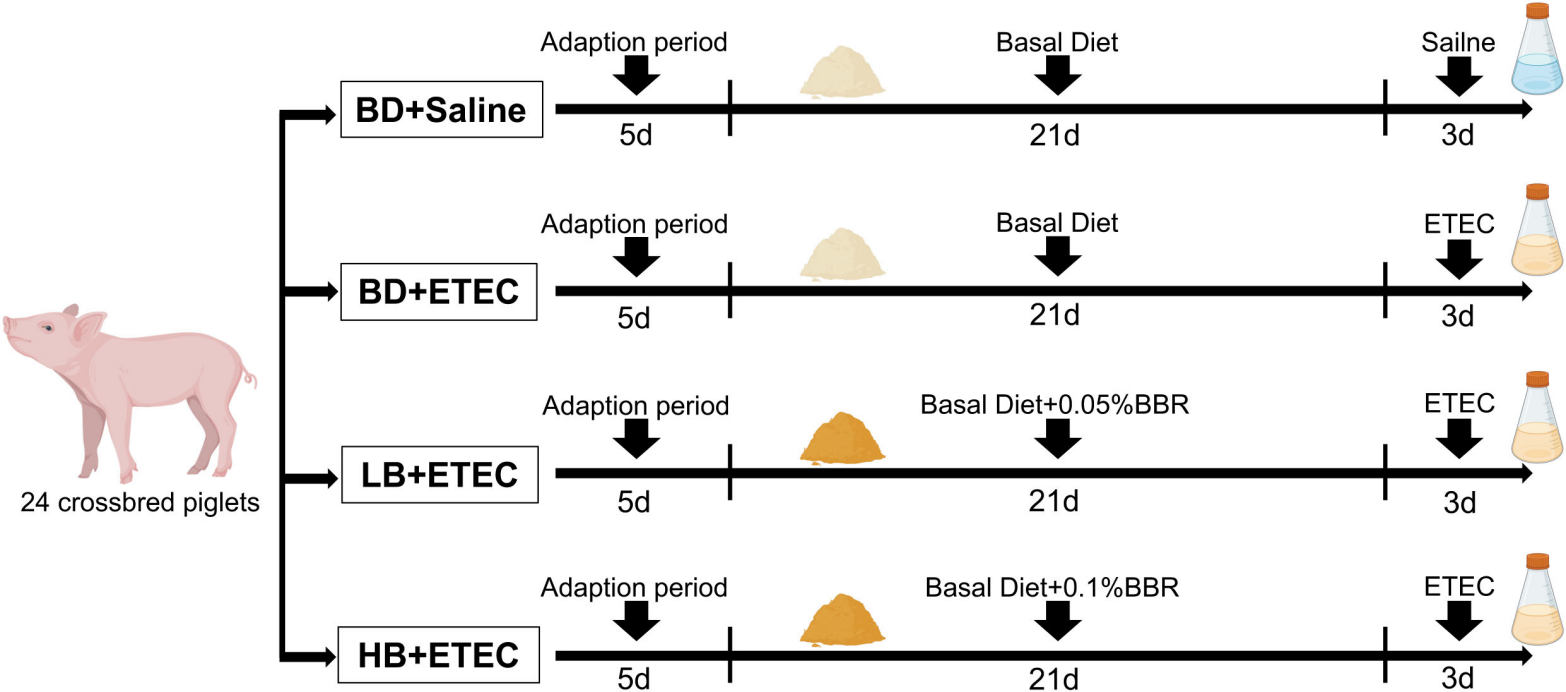
Animal experimentation design. BD, basal diet; LB, basal diet with 0.05% berberine; HB, basal diet with 0.1% berberine.

For this study, 24 crossbred pigs (Duroc × Landrace × Large Yorkshire) were purchased from a local farm. Pigs were weaned at 21 days of age and placed in a controlled environment following previously described procedures (21, 22). All pigs had free access to water and feed and received the same basic diet (23). The weaned piglets were randomly distributed into the following four experimental groups: a BD+Saline group (basal diet with saline orally administered to piglets), a BD+ETEC group (basal diet with ETEC orally administered to piglets at 1 × 10^9^ CFU per pig), a LB+ETEC group (basal diet with 0.05% [low dose] berberine, with ETEC orally administered), and a HB+ETEC group (basal diet with 0.1% [high dose] berberine, with ETEC orally administered). The feeding adaptation period lasted for 5 days and the experiment period lasted for 21 days. At the end of the feeding period, piglets in the BD+ETEC, LB+ETEC, and HB+ETEC groups received 10 mL of ETEC (1 × 10^8^ CFU/mL) orally, while piglets in the BD+Saline group received 10 mL of saline for 3 consecutive days (**Figure 1**). The experimental diets were provided in meal form and berberine chloride hydrate (purity ≥ 98%) was purchased from Aladdin Reagent Co., Ltd (Shanghai, China) (22). The composition and nutrient contents of the experimental diets are detailed in **Supplementary Table S1**.

### 2.4 Growth performance and diarrheal incidence in piglets

The fasting body weight (BW) of each pig before feeding was measured at 06:00 am on days 1 and 25 of the experimental period. The average daily gain (ADG), average daily feed intake (ADFI), and feed-to-gain ratio (F: G) of the piglets were calculated according to the feeding amount and residual feed records. Diarrheal incidence was monitored twice a day at 08:00 am and 14:00 pm using a scoring system, as previously described. Briefly, feces were categorized as normal (solid feces; score: 0), slight diarrhea (soft and loose feces; score: 1), moderate diarrhea (semi-liquid feces; score: 2), and severe diarrhea (liquid and unformed feces; score: 3). Diarrhea was defined as a score of 2 or 3 per day and the daily diarrheal incidence was calculated as follows: diarrheal rate (%) = (number of pigs with diarrhea × days with diarrhea)/(total number of pigs × experimental days) (24, 25).

### 2.5 Sample collection

On day 25, the piglets were humanely euthanized using sodium pentobarbital (40 mg/kg BW) and dissected. Samples of the small intestine (middle parts of the duodenum, jejunum, and ileum) were stored at −80°C and prepared for RNA extraction. These small intestinal samples were used for the analysis of inflammatory factors and oxidative stress indicators. Colonic digesta samples were gathered for metagenomic analysis.

### 2.6 Determination of intestinal inflammatory cytokines, oxidation markers, and antioxidative enzymes

Determination of total antioxidant capacity (T-AOC) (Cat. No A015-2-1) and total superoxide dismutase (T-SOD) (Cat. No A001-3-2), glutathione peroxidase (GSH-Px) (Cat. No A005-1-2), catalase (CAT) (Cat. No A007-2-1) activities, and malondialdehyde (MDA) (Cat. No A003-1-2) contents in the small intestinal (duodenum, jejunum, ileum) tissue homogenates were assayed using the respective kits (Nanjing Jiancheng Bioengineering Institute, Jiangsu, China). The contents of 8-hydroxy-2 deoxyguanosine (8-OHdG) (Cat. No DKM2508A), tumor necrosis factor-alpha (TNF-α) (Cat. No DKM2523A), interleukin (IL)-1β (Cat. No DKM2562A), IL-6 (Cat. No DKM2558A), transforming growth factor-beta (TGF-β) (Cat. No DKM2522A), and IL-10 (Cat. No DKM2565A) were assessed using commercially available ELISA kits (Beijing Dakome Technology Company, Beijing, China) following the manufacturer’s protocols (21).

### 2.7 Reverse transcription-quantitative PCR

RT-qPCR was performed as previously described (21). Total RNA was extracted from the duodenal, jejunal, and ileal tissues using the RNA Easy Fast Tissue/Cell Kit (TIANGEN, DP451, Beijing, China) following the guidelines provided by the manufacturer. The entire RNA sample was reverse-transcribed into cDNA using the HiScript III 1st Strand cDNA Synthesis Kit (+gDNA eliminator) (Vazyme, R312, Nanjing, China). Quantitative PCR was performed according to the protocols described in our previous study (21). Primers were designed using Primer 5.0 and were synthesized by Shanghai Sanggong Biotechnology Co., Ltd (Shanghai, China). The primer sequences are listed in **Supplementary Table S2**. The Ct values of the target genes were normalized based on the geometric mean Ct values of β-actin and GAPDH. Subsequently, the 2^-ΔΔCt^ method was used to determine the relative mRNA expression of the target genes (21).

### 2.8 Metagenomic sequencing

Metagenomic sequencing was performed as previously described (22). Metagenome sequencing data were deposited in the Sequence Read Archive (SRA) database (https://www.ncbi.nlm.nih.gov/sra) of NCBI under the BioProject accession numbers PRJNA1110045 and SAMN41316941 and the SRA project accession numbers SRR 29048499 to 29048514. The Log2 Fold-Change (FC) was calculated using Microsoft Excel. The screening criteria for KEGG and eggNOG analysis were log2FC > 1.0 or < −1.0 and an adjusted *P*-value of <0.05.

### 2.9 Statistical analysis

After testing for normality and performing the necessary transformations, data between groups were compared using one-way ANOVA. The non-parametric Kruskal-Wallis test was used to process the relative abundance of intestinal microbial communities and data that did not follow a normal distribution. Correlation analysis was performed using Pearson’s correlation test. *P*<0.05 was considered statistically significant. All statistical analyses were performed using SPSS (v.26.0) and graphs were generated using GraphPad Prism (v.10.0) software (21, 25).

## 3 Results

### 3.1 Growth performance and diarrheal incidence

The diarrheal rate of piglets in the BD+ETEC group was significantly higher than that in the BD+Saline group (*P*<0.05). Compared with the BD+ETEC group, the final BW of piglets in the HB+ETEC group was significantly increased, as were the ADG in the LB+ETEC and HB+ETEC groups and the ADFI in the LB+ETEC group (*P*<0.05); in contrast, the diarrheal rate in the LB+ETEC and HB+ETEC groups was significantly decreased (*P*<0.01) (**Table 1**).

**Table 1:** Effects of dietary berberine on growth performance and diarrhea rate of weaned piglets infected with ETEC.

Measure	Experimental diets				SEM	*P*-Value
	BD+Saline	BD+ETEC	LB+ETEC	HB+ETEC		
Initial BW, kg	6.03	5.31	5.46	5.99	0.18	0.43
Final BW, kg	9.75	9.34^b^	11.16^ab^	12.68^a^	0.50	<0.05
ADG, g	168.84	168.35^b^	283.48^a^	286.52^a^	19.11	<0.05
ADFI, g	421.25	433.87^b^	795.11^a^	579.05^ab^	43.40	<0.05
F: G ratio, g/g	2.61	2.68	2.62	2.25	0.12	0.22
Diarrhea rate, %	6.57	7.33^a*^	2.61^b^	2.38^b^	0.42	<0.01

^*^BD+Saline vs BD+ETEC; ^a,b^BD+ETEC vs LB+ETEC vs HB+ETEC; ^a,b^Values with different superscripts in the row indicate significant differences (*P*<0.05).

### 3.2 Proinflammatory and anti-inflammatory factors

**Figure 2:**
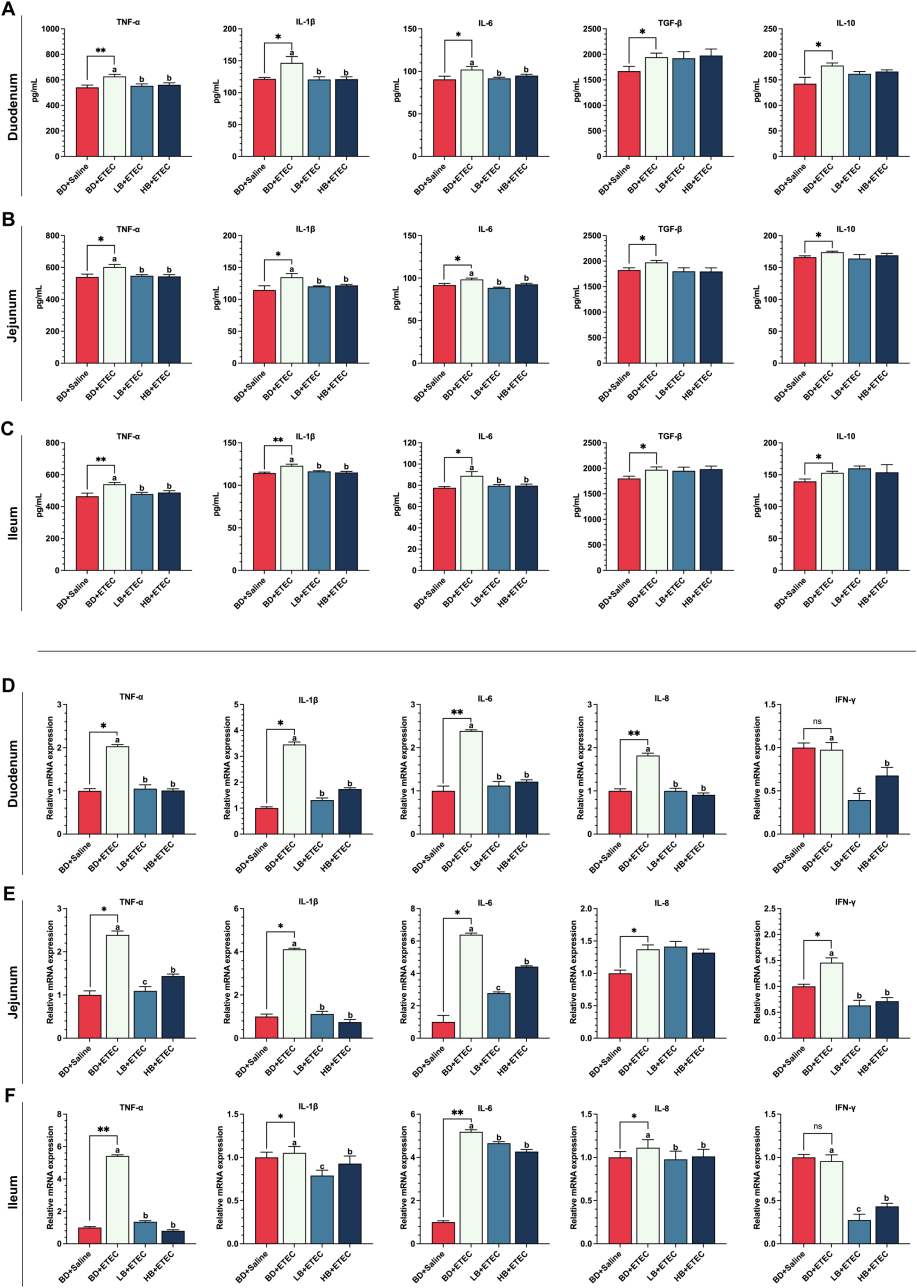
The effects of dietary berberine on the contents of proinflammatory factors, anti-inflammatory factors, and the expression of inflammation-related genes in the small intestine of weaned piglets infected with enterotoxigenic *Escherichia coli* (ETEC). The contents of proinflammatory factors (TNF-α, IL-1β, IL-6) and anti-inflammatory factors (TGF-β, IL-10) in the duodenum **(A)**, jejunum **(B)**, and ileum **(C)** in the different treatment groups. The expression of inflammation-related genes (TNF-α, IL-1β, IL-6, IL-8, IFN-γ) in the duodenum **(D)**, jejunum **(E)**, and ileum **(F)** in the different treatment groups. ^*^BD+Saline *vs*. BD+ETEC, ^*^ *P*<0.05, ^**^ *P*<0.01; ^a,b,c^BD+ETEC *vs*. LB+ETEC *vs*. HB+ETEC; BD+Saline, a basal diet with saline orally administered to piglets; BD+ETEC, a basal diet with ETEC orally administered to piglets; LB+ETEC, a basal diet with 0.05% berberine, ETEC orally administered to piglets; HB+ETEC, a basal diet with 0.1% berberine, ETEC orally administered to piglets.

Compared with those in the BD+Saline group, the contents of TNF-α, IL-1β, IL-6, TGF-β, and IL-10 in the duodenum (**Figure 2A**), jejunum (**Figure 2B**), and ileum (**Figure 2C**) were significantly increased in the BD+ETEC group (*P*<0.05). Compared with the BD+ETEC group, the contents of TNF-α, IL-1β, and IL-6 in the duodenum (**Figure 2A**), jejunum (**Figure 2B**), and ileum (**Figure 2C**) were significantly decreased in the LB+ETEC and HB+ETEC groups (*P*<0.05).

### 3.3 The expression of inflammatory-related genes in the small intestine

Compared with the BD+Saline group, the expression of the genes encoding TNF-α, IL-1β, IL-6, and IL-8 in the duodenum (**Figure 2D**), jejunum (**Figure 2E**), and ileum (**Figure 2F**) were significantly increased in the BD+ETEC group (*P*<0.05). Additionally, the expression levels of the genes coding for TNF-α, IL-1β, IL-6, IL-8, and IFN-γ in the duodenum (**Figure 2D**) and ileum (**Figure 2F**) were significantly higher in the BD+ETEC group than in the LB+ETEC and HB+ETEC groups (*P*<0.05), whereas those of genes encoding TNF-α, IL-1β, IL-6, and IFN-γ in the jejunum (**Figure 2E**) were significantly decreased (*P*<0.05).

### 3.4 The TLR4/MyD88/NF-κB signaling pathway in the small intestine

**Figure 3:**
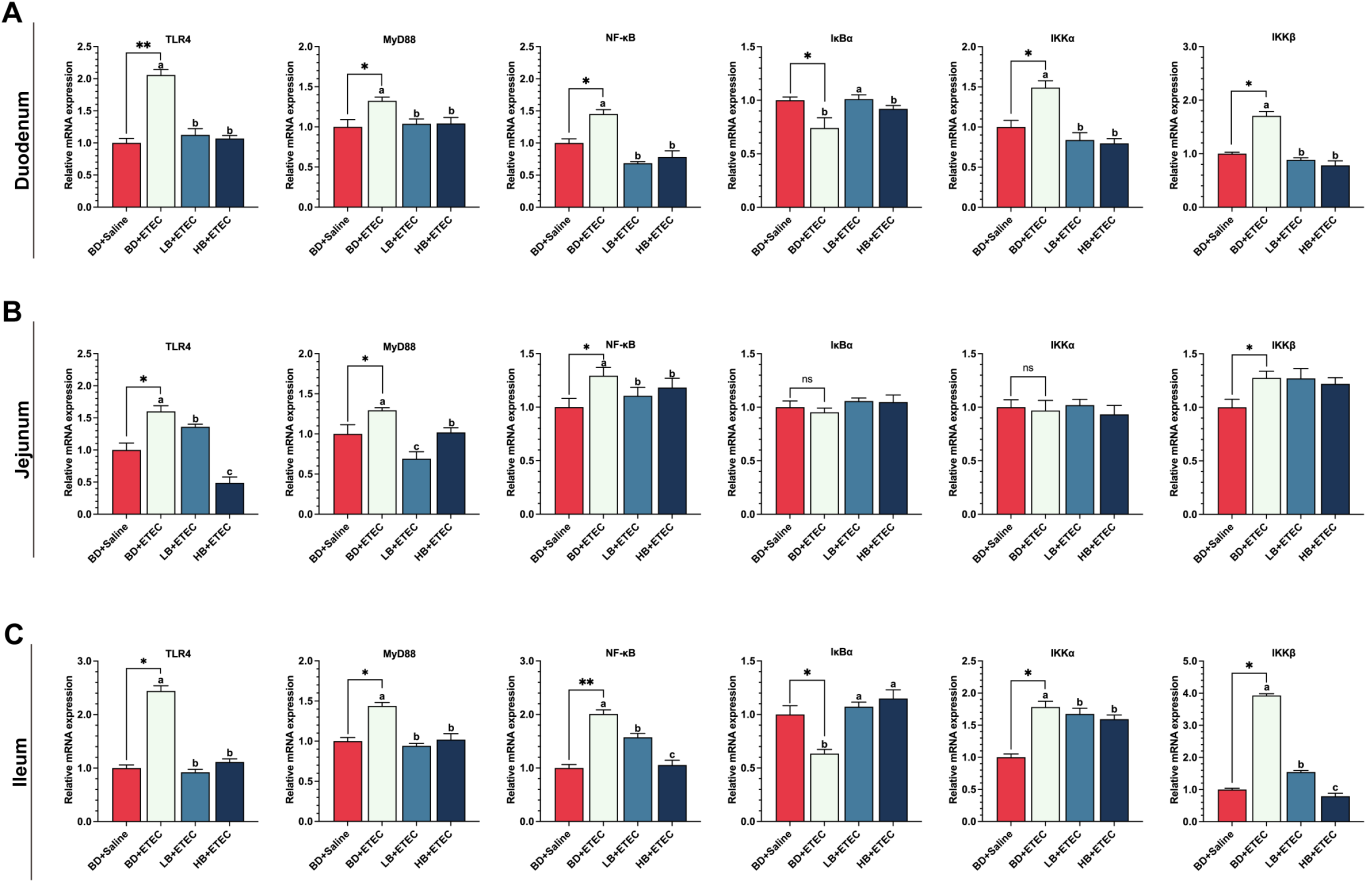
The effects of dietary berberine on the TLR4/MyD88/NF-κB signaling pathway in weaned piglets infected with enterotoxigenic *Escherichia coli* (ETEC). The expression of genes in the TLR4/MyD88/NF-κB signaling pathway (TLR4, MyD88, NF-κB, IκBα, IKKα, IKKβ) in the duodenum **(A)**, jejunum **(B)**, and ileum **(C)** in the different treatment groups. ^*^BD+Saline *vs*. BD+ETEC, ^*^ *P*<0.05, ^**^ *P*<0.01; ^a,b,c^BD+ETEC *vs*. LB+ETEC *vs*. HB+ETEC; BD+Saline, a basal diet with saline orally administered to piglets; BD+ETEC, a basal diet with ETEC orally administered to piglets; LB+ETEC, a basal diet with 0.05% berberine, ETEC orally administered to piglets; HB+ETEC, a basal diet with 0.1% berberine, ETEC orally administered to piglets.

The expression levels of the TLR4, MyD88, NF-κB, IKKα, and IKKβ encoding genes were significantly higher in the duodenum (**Figure 3A**) and ileum (**Figure 3C**) of the BD+ETEC group compared with those in the BD+Saline group (*P*<0.05); except for IKKα, a similar expression trend was observed in the jejunum of the BD+ETEC group (**Figure 3B**) (*P*<0.05). In contrast, the expression of the IκBα gene was significantly decreased in the duodenum (**Figure 3A**) and ileum (**Figure 3C**) in the BD+ETEC group (*P*<0.05). Compared with the BD+ETEC group, the expression levels of the genes encoding TLR4, MyD88, NF-κB, IKKα, and IKKβ were significantly decreased in the duodenum (**Figure 3A**) and ileum (**Figure 3C**) in the LB+ETEC and HB+ETEC groups (*P*<0.05); similarly, the expression of the TLR4, MyD88, and NF-κB encoding genes was significantly decreased (*P*<0.05) in the jejunum of piglets in the LB+ETEC and HB+ETEC groups (**Figure 3B**), while that of the gene encoding IκBα was significantly increased (*P*<0.05) in the duodenum (**Figure 3A**) and ileum (**Figure 3C**) in the LB+ETEC and HB+ETEC groups.

### 3.5 The levels of oxidation markers and antioxidant enzyme activities in the small intestine

**Figure 4:**
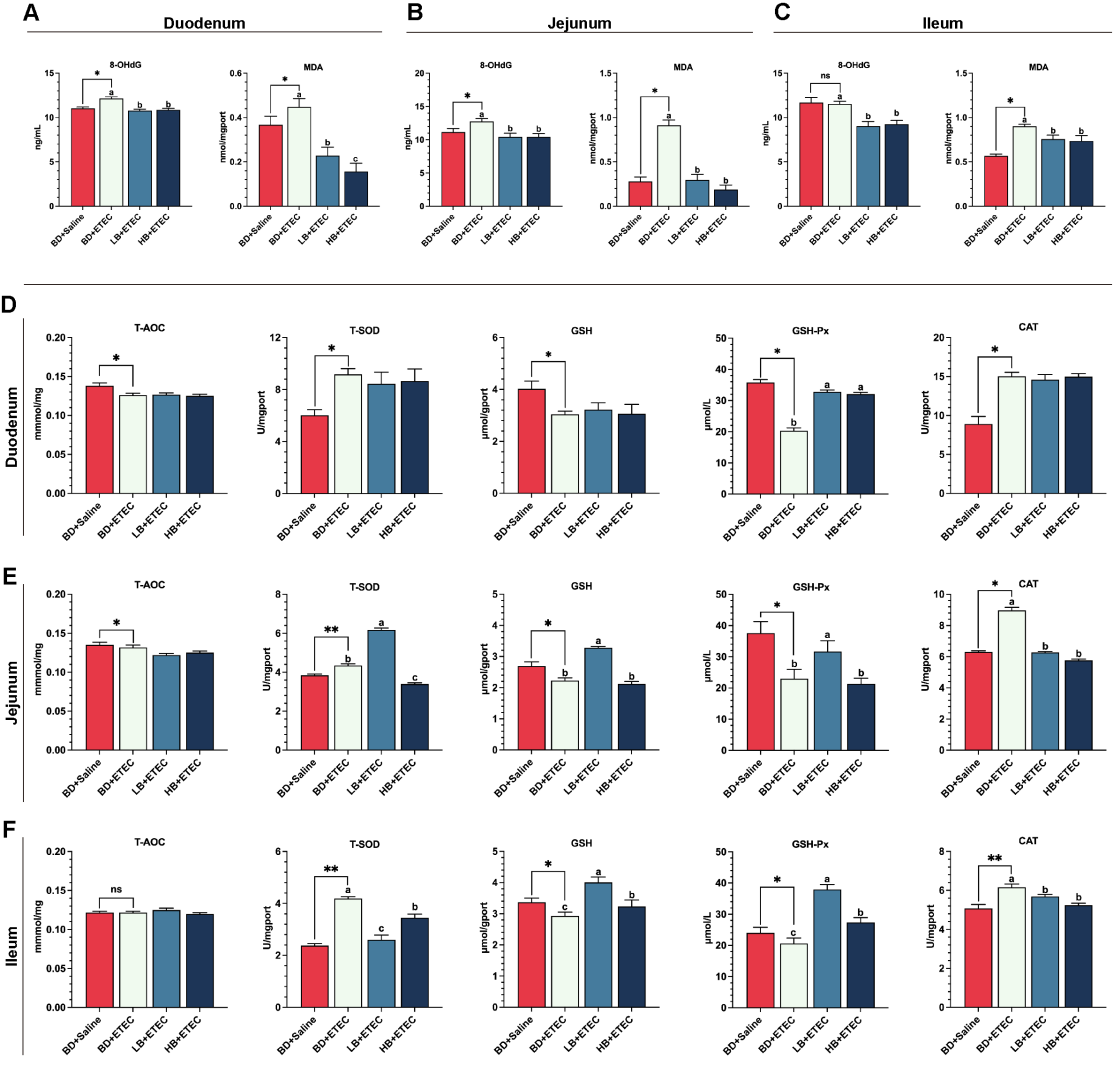
The effects of dietary berberine on oxidation markers and antioxidant enzyme activities in the small intestine of weaned piglets infected with enterotoxigenic *Escherichia coli* (ETEC). The contents of oxidation markers (8-OHdG, MDA) in the duodenum **(A)**, jejunum **(B)**, and ileum **(C)** in the different treatment groups. The T-AOC and the contents of antioxidant enzymes (T-SOD, GSH, GSH-Px, CAT) in the duodenum **(D)**, jejunum **(E)**, and ileum **(F)** in the different treatment groups. ^*^ *P*<0.05, ^**^ *P*<0.01; ^a,b,c^BD+ETEC *vs*. LB+ETEC *vs*. HB+ETEC; BD+Saline, a basal diet with saline orally administered to piglets; BD+ETEC, a basal diet with ETEC orally administered to piglets; LB+ETEC, a basal diet with 0.05% berberine, ETEC orally administered to piglets; HB+ETEC, a basal diet with 0.1% berberine, ETEC orally administered to piglets.

Compared with the BD+Saline group, the contents of 8-OHdG and MDA in the duodenum (**Figure 4A**) and jejunum (**Figure 4B**) were significantly increased (*P*<0.05) in the BD+ETEC group, as were the ileal MDA contents in the BD+ETEC group (*P*<0.05) (**Figure 4C**). Compared with the BD+ETEC group, the contents of 8-OHdG and MDA in the duodenum (**Figure 4A**), jejunum (**Figure 4B**), and ileum (**Figure 4C**) were significantly decreased (*P*<0.05) in the LB+ETEC and HB+ETEC groups. T-SOD and CAT contents in the duodenum (**Figure 4D**), jejunum (**Figure 4E**), and ileum (**Figure 4F**) were significantly higher in the BD+ETEC group than in the BD+Saline group (*P*<0.05). Additionally, the activities of T-AOC and the GSH, and GSH-Px in the duodenum (**Figure 4D**) and jejunum (**Figure 4E**), as well as the GSH and GSH-Px activities in the ileum (**Figure 4F**), were significantly lower (*P*<0.05) in the BD+ETEC group than in the BD+Saline group. Compared with the BD+ETEC group, the activities of GSH-Px in the duodenum (**Figure 4D**) and those of GSH and GSH-Px in the ileum (**Figure 4F**) were significantly increased (*P*<0.05) in the LB+ETEC and HB+ETEC groups, as were those of T-SOD, GSH, and GSH-Px in the jejunum (**Figure 4E**) in the LB+ETEC group (*P*<0.05). Finally, the CAT activity in the jejunum (**Figure 4E**) and the T-SOD and CAT activities in the ileum (**Figure 4F**) were significantly lower (*P*<0.05) in the LB+ETEC and HB+ETEC groups than in the BD+ETEC group.

### 3.6 The expression of the *Nrf2* gene, antioxidant-related genes, and detoxifying enzyme-related genes in the small intestine

**Figure 5:**
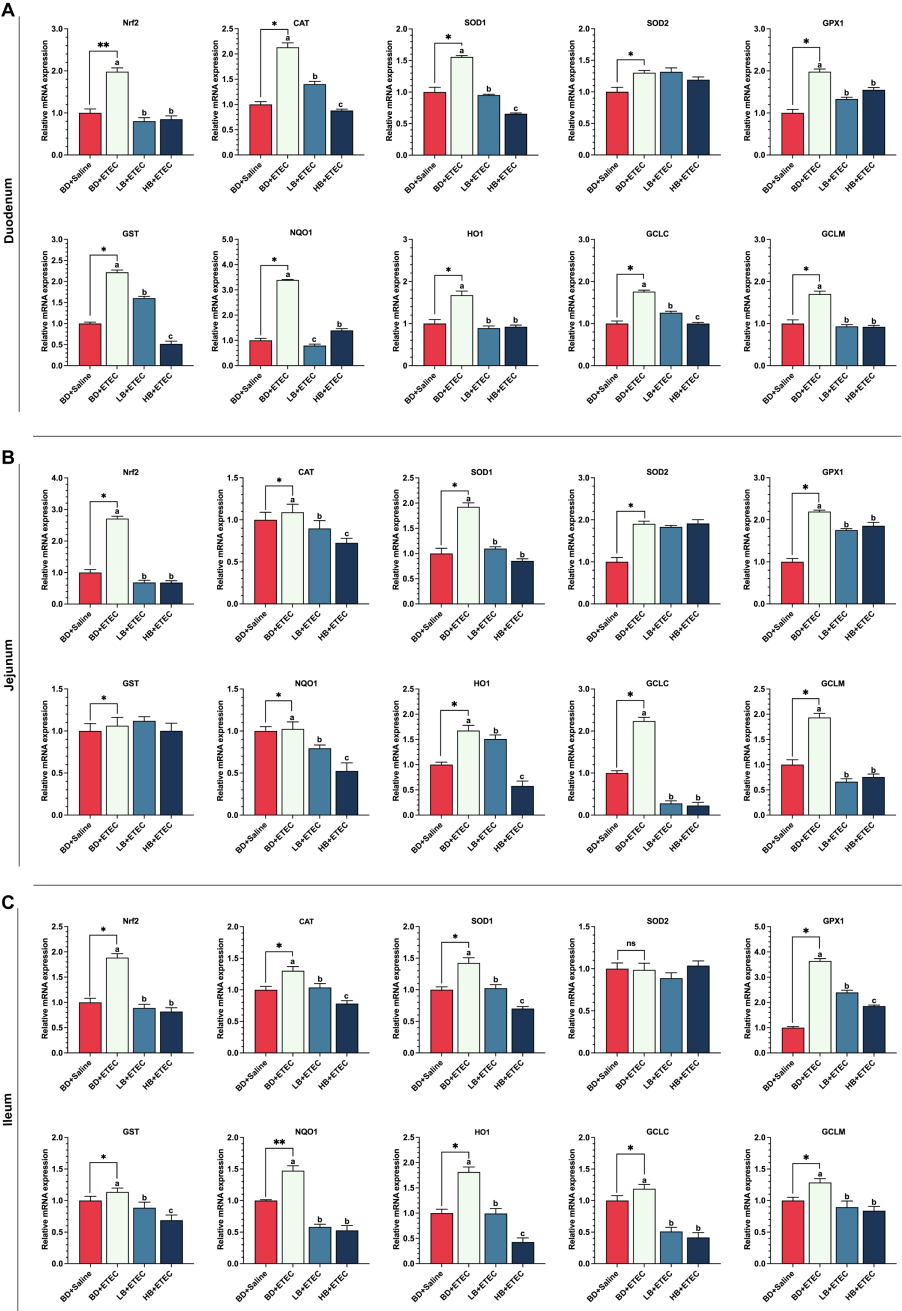
The effects of dietary berberine on the Nrf2 signaling pathway and antioxidant- and detoxifying enzyme-related genes in the small intestine of weaned piglets infected with enterotoxigenic *Escherichia coli* (ETEC). The expression of Nrf2, antioxidant coding genes (CAT, SOD1, GPX1, GST), and phase II detoxifying enzyme coding genes (NQO1, HO-1, GCLC, GCLM) in the duodenum **(A)**, jejunum **(B)**, and ileum **(C)** in the different treatment groups. ^*^ *P*<0.05, ^**^ *P*<0.01; ^a,b,c^BD+ETEC *vs*. LB+ETEC *vs*. HB+ETEC; BD+Saline, a basal diet with saline orally administered to piglets; BD+ETEC, a basal diet with ETEC orally administered to piglets; LB+ETEC, a basal diet with 0.05% berberine, ETEC orally administered to piglets; HB+ETEC, a basal diet with 0.1% berberine, ETEC orally administered to piglets.

Compared with the BD+Saline group, the expression levels of the genes encoding Nrf2, CAT, SOD1, SOD2, GPX1, GST, NQD1, HO1, GCLC, and GCLM in the duodenum (**Figure 5A**) and ileum (**Figure 5B**), as well as those of the genes coding for Nrf2, CAT, SOD1, GPX1, GST, NQD1, HO1, GCLC, and GCLM in the ileum (**Figure 5C**) were significantly upregulated in the BD+ETEC group (*P*<0.05). Additionally, the expression levels of the Nrf2, CAT, SOD1, GPX1, GST, NQD1, HO1, GCLC, and GCLM coding genes in the duodenum (**Figure 5A**) and ileum (**Figure 5B**), as well as those of genes encoding Nrf2, CAT, SOD1, GPX1, NQD1, HO1, GCLC, and GCLM in the ileum (**Figure 5C**), were significantly lower (*P*<0.05) in the LB+ETEC and HB+ETEC groups than in the BD+Saline group.

### 3.7 Taxonomic configurations of the intestinal microbiota and the intestinal microbiota structure

**Figure 6:**
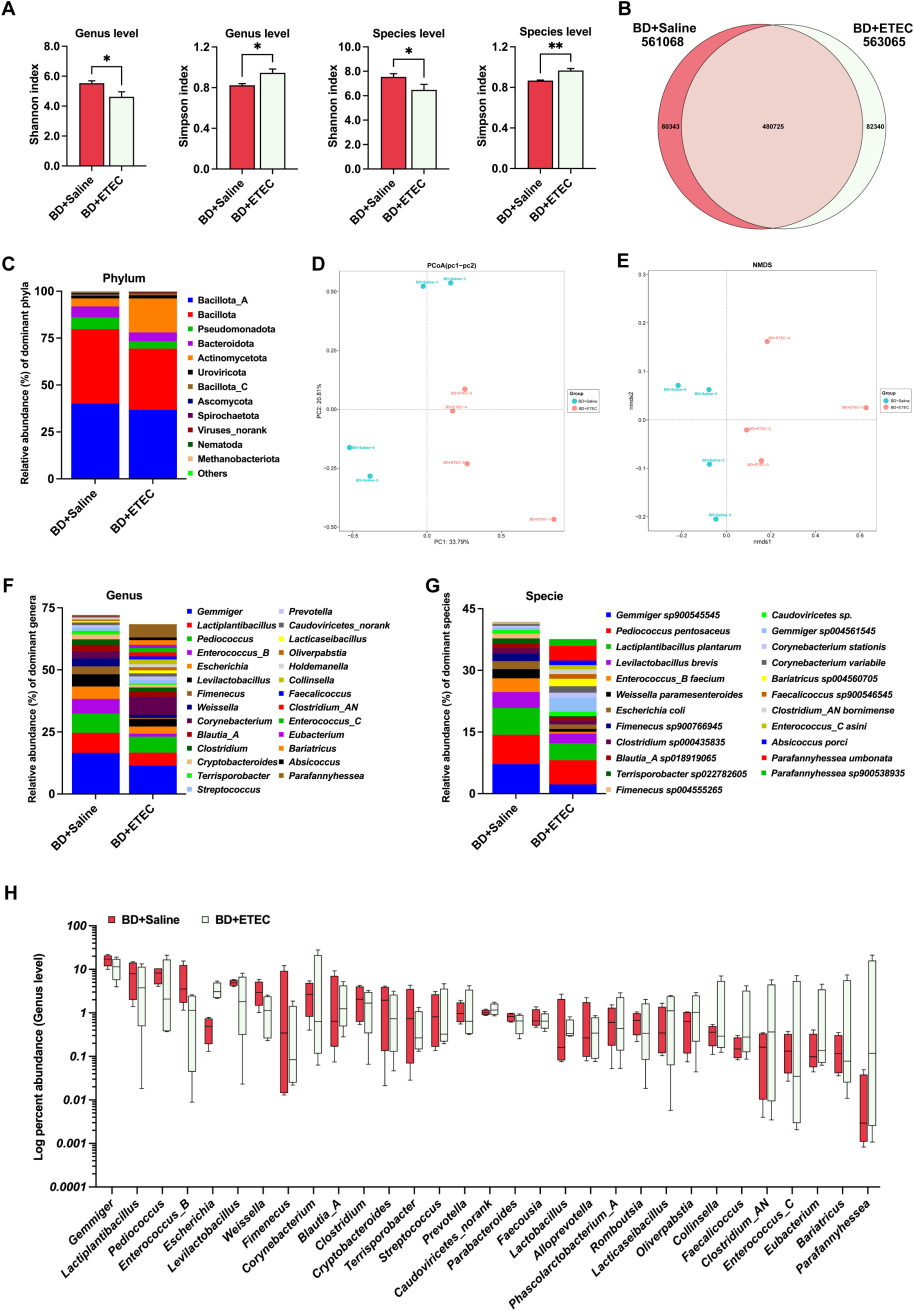
The effects of enterotoxigenic *Escherichia coli* (ETEC) infection on the intestinal microbiota of weaned piglets. **(A)** The diversity (Shannon and Simpson indexes) of the intestinal microbiota of weaned piglets (genus and species levels). **(B)** A Venn diagram of the species in the different treatment groups. **(D)** Principal Coordinates Analysis (PCoA). **(E)** Nonmetric multidimensional scaling (NMDS) analysis. The composition and structure of the intestinal microbiota of weaned piglets (relative abundance > 1%) at the phylum level **(C)**, family level **(F)**, and genus level **(G)**. **(H)** Relative bacterial abundance at the genus level between the BD+Saline and BD+ETEC groups (*P*<0.05). ^*^BD+Saline *vs*. BD+ETEC, ^*^ *P*<0.05, ^**^ *P*<0.01; BD+Saline, basal diet with saline orally administered to piglets; BD+ETEC, basal diet with ETEC orally administered to piglets.

**Figure 7:**
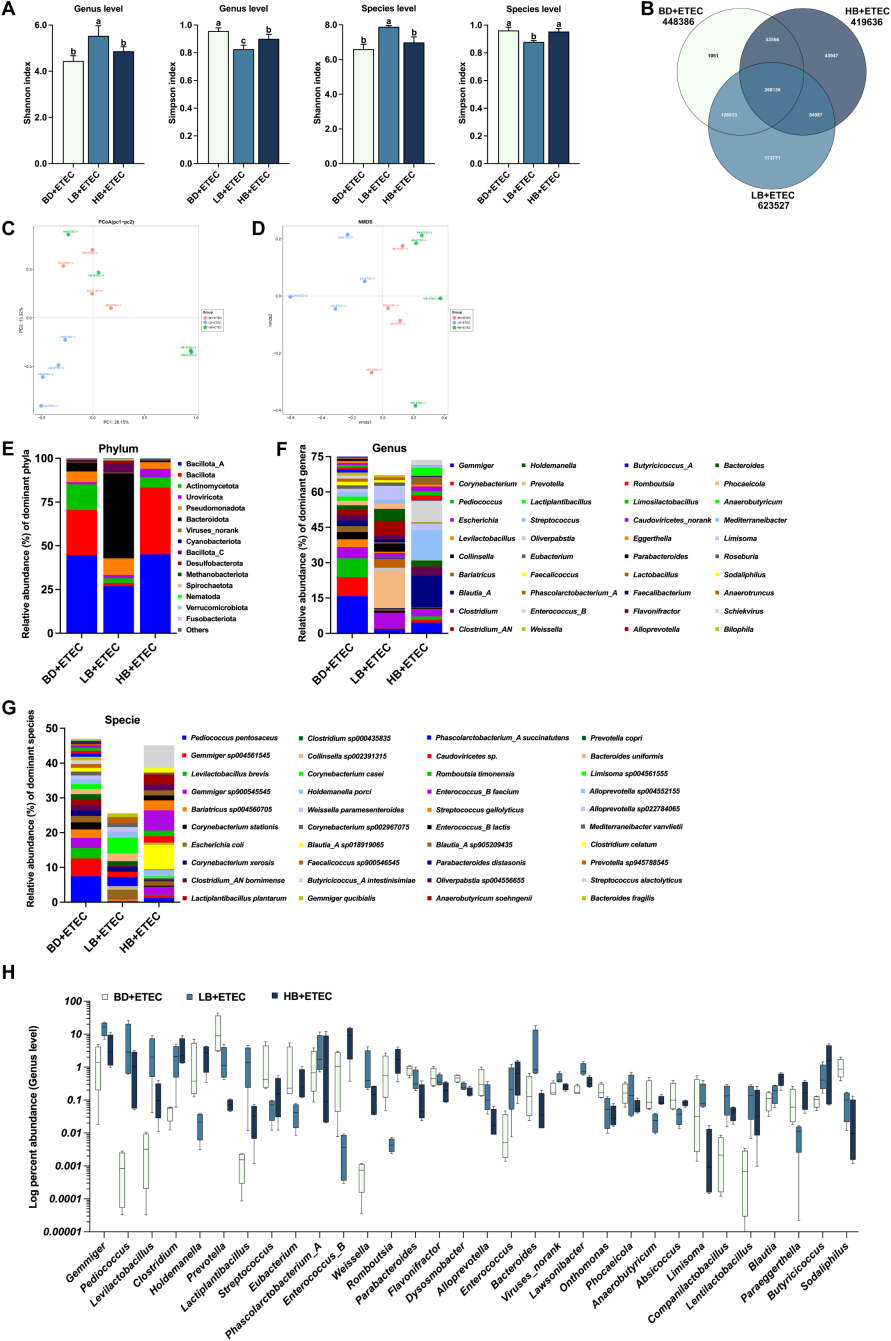
The effects of dietary berberine on the intestinal microflora in weaned piglets infected with enterotoxigenic *Escherichia coli* (ETEC). **(A)** The diversity (Shannon and Simpson indexes) of the intestinal microbiota of weaned piglets (genus and species level). **(B)** A Venn diagram of the species in the different treatment groups. **(C)** Principal Coordinates Analysis (PCoA). **(D)** Nonmetric multidimensional scaling (NMDS) analysis. The composition and structure of the intestinal microbiota of weaned piglets (relative abundance > 1%) at the phylum level **(E)**, family level **(F)**, and genus level **(G)**. **(H)** Relative bacterial abundance at the genus level among the BD+ETEC, LB+ETEC, and HB+ETEC groups (*P*<0.05). ^a,b,c^BD+ETEC *vs*. LB+ETEC *vs*. HB+ETEC. BD+ETEC, a basal diet with ETEC orally administered to piglets; LB+ETEC, a basal diet with 0.05% berberine, ETEC orally administered to piglets; HB+ETEC, a basal diet with 0.1% berberine, ETEC orally administered to piglets.

To investigate the impact of ETEC infection and berberine intervention on the intestinal microbiota, metagenomic sequencing was conducted. The microbial structure profiles are shown in **Figures 6** and **7**. Compared with the BD+Saline group, the Shannon index was decreased while the Simpson index was increased in the BD+ETEC group (*P*<0.05) (**Figure 6A**). The Venn profile indicated that 480,725 microbial species were shared between the two groups, with 80,343 and 82,340 microbial species being unique to the BD+Saline and BD+ETEC groups, respectively (**Figure 6B**). Based on Bray-Curtis distances, Principal Coordinates Analysis (PCoA) and nonmetric multidimensional scaling (NMDS) analysis demonstrated that microbial community structure differed significantly between the BD+Saline and BD+ETEC groups (**Figures 6D, E**). Additionally, discriminatory features were observed in microbial relative abundance at the phylum, genus, and species levels; 12 phyla (**Figure 6C**), 27 genera (**Figure 6F**), and 23 species (**Figure 6G**) had a relative abundance greater than 1% and were identified as dominant. Compared with the BD+Saline group, the BD+ETEC group exhibited a noteworthy increase in the relative abundance of *Escherichia*, *Blautia*_A, *Caudoviricetes*_norank, *Lactobacillus*, *Alloprevotella*, *Lacticaseibacillus*, *Faecalicoccus*, *Clostridium*_AN, *Eubacterium*, and *Parafannyhessea*. Conversely, a significant decrease was observed in the relative abundance of *Gemmiger*, *Lactiplantibacillus*, *Pediococcus*, *Enterococcus*_B, *Levilactobacillus*, *Weissella*, *Fimenecus*, *Corynebacterium*, *Clostridium*, *Cryptobacteroides*, *Terrisporobacter*, *Streptococcus*, *Prevotella*, *Parabacteroides*, *Faecousia*, *Phascolarctobacterium*_A, *Romboutsia*, *Oliverpabstia*, *Collinsella*, *Enterococcus*_C, and *Bariatricus* in the BD+ETEC group compared with that in the BD+Saline group (*P*<0.05) (**Figure 6H**).

Compared with the BD+ETEC group, the Shannon index was increased in the LB+ETEC group at both the genus and species levels (*P*<0.05); meanwhile, the Simpson index was decreased in both the LB+ETEC and HB+ETEC groups at the genus level, as well as in the LB+ETEC group at the species level (*P*<0.05) (**Figure 7A**). As shown in the Venn diagram in **Figure 7B**, a total of 268,136 microbial species were common to both groups, with 1,051, 173,771, and 43,947 microbial species being unique to the BD+ETEC, LB+ETEC, and HB+ETEC groups, respectively. PCoA and NMDS analysis, based on Bray-Curtis distances, revealed significant differences in microbial community structure between the LB+ETEC group and the other groups (**Figures 7C, D**). The 15 phyla (**Figure 7E**), 40 genera (**Figure 7F**), and 40 species (**Figure 7G**) with a relative abundance greater than 1% were found to be dominant. Compared with the BD+ETEC group, the LB+ETEC and HB+ETEC groups showed a significant increase in the relative abundance of *Gemmiger*, *Pediococcus*, *Levilactobacillus*, *Clostridium*, *Lactiplantibacillus*, *Phascolarctobacterium*_*A*, *Weissella*, *Enterococcus*, *Lawsonibacter*, *Companilactobacillus*, *Lentilactobacillus*, *Blautia*, and *Butyricicoccus*, whereas the relative abundance of *Prevotella*, *Streptococcus*, *Parabacteroides*, *Flavonifractor*, *Dysosmobacter*, *Alloprevotella*, *Onthomonas*, *Phocaeicola*, *Absicoccus*, and *Sodaliphilus* displayed a significant decrease (*P*<0.05) (**Figure 7H**).

### 3.8 Metagenomic analysis of the intestinal microbiota

**Figure 8:**
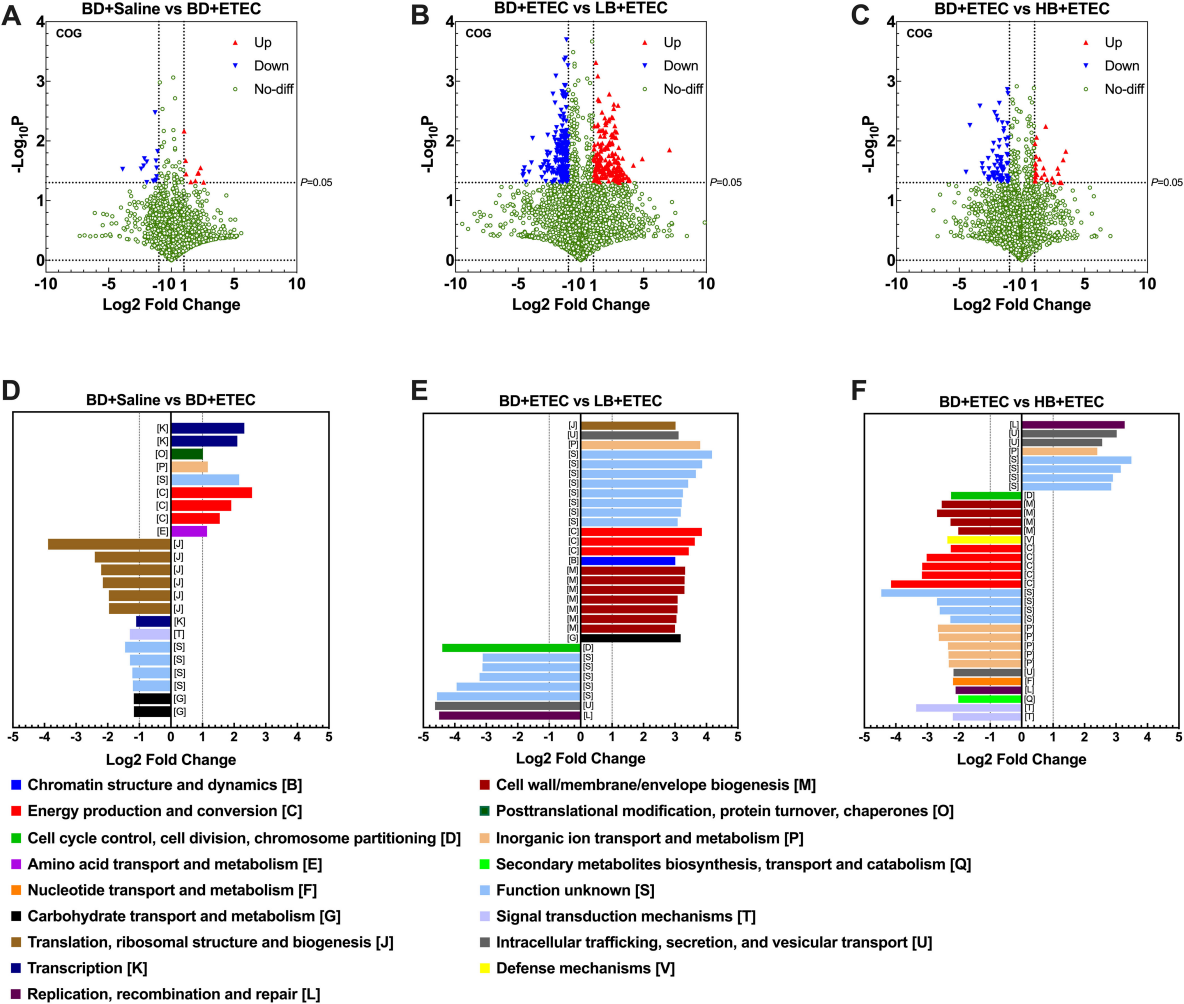
Clusters of orthologous groups (COG) functional annotation of the metagenomic sequencing data. A volcano plot showing the distribution of the identified functional COGs between the BD+Saline and BD+ETEC (enterotoxigenic *Escherichia coli*) groups **(A)**, the BD+ETEC and LB+ETEC groups **(B)**, and the BD+ETEC and HB+ETEC groups **(C)**. The biologically significant differences in functional clusters of orthologous groups of protein categories between the BD+Saline and BD+ETEC groups **(D)**, the BD+ETEC and LB+ETEC groups **(E)**, and the BD+ETEC and HB+ETEC groups **(F)**. BD+Saline, a basal diet with saline orally administered to piglets; BD+ETEC, a basal diet with ETEC orally administered to piglets; LB+ETEC, a basal diet with 0.05% berberine, ETEC orally administered to piglets; HB+ETEC, a basal diet with 0.1% berberine, ETEC orally administered to piglets.

Next, we identified the biologically significant differences in functional clusters of orthologous groups (COG) of protein categories between each two groups. The volcano plots in **Figures 8A–C** depict the distribution of the identified functional COGs between each two groups. A total of 4,514 functional COGs were found between the BD+Saline and BD+ETEC groups. Among these, the relative abundance of 9 COGs was significantly upregulated (*P*<0.05, log2FC ≥ 1), while that of 14 COGs was significantly downregulated (*P*<0.05, log2FC ≤ −1) (**Figure 8A**). Of these COG categories, six ([K], [O], [P], [S], [C], [E]) were upregulated and enriched in the BD+ETEC group, and five ([J], [K], [T], [S], [G]) were downregulated and enriched in the BD+Saline group (**Figure 8D**). When comparing the BD+ETEC and LB+ETEC groups, out of 4,287 functional COGs analyzed, 184 showed significant upregulation and 201 showed significant downregulation (**Figure 8B**). Among these COG categories, eight ([J], [U], [P], [S], [C], [B], [M], [G]) were found to be upregulated and enriched in the LB+ETEC group, while four ([D], [S], [U], [L]) were downregulated and enriched in the BD+ETEC group (**Figure 8E**). When comparing the BD+ETEC and HB+ETEC groups, out of 3,810 functional COGs analyzed, 26 were significantly upregulated while 73 were significantly downregulated (**Figure 8C**). Among these COG functional categories, 4 ([L], [U], [P], [S]) were upregulated and enriched in the HB+ETEC group, and 11 ([D], [M], [V], [C], [S], [P], [U], [F], [L], [Q], [T]) were downregulated in the BD+ETEC group (**Figure 8F**).

**Figure 9:**
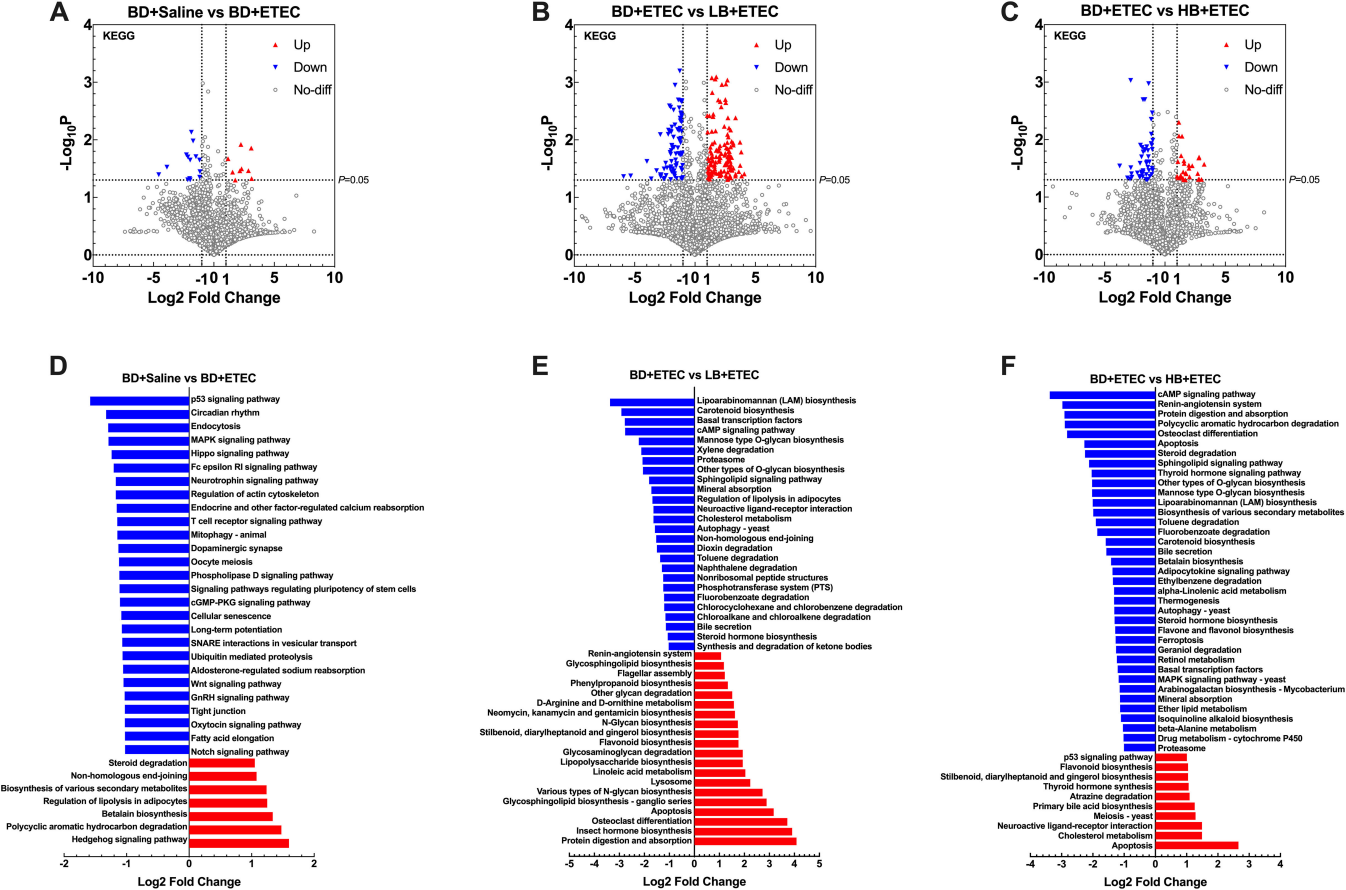
Kyoto Encyclopedia of Genes and Genomes (KEGG) analyses of the metagenomic sequencing data at the functional level. A volcano plot showing the distribution of the identified functional KEGG pathways that differed between the BD+Saline and BD+ETEC groups **(A)**, the BD+ETEC and LB+ETEC groups **(B)**, and the BD+ETEC and HB+ETEC groups **(C)**. Differential analysis of the metagenomic microbial function profiling in KEGG level 2 functional categories between the BD+Saline and BD+ETEC groups **(D)**, the BD+ETEC and LB+ETEC groups **(E)**, and the BD+ETEC and HB+ETEC groups **(F)**. BD+Saline, a basal diet with saline orally administered to piglets; BD+ETEC, a basal diet with ETEC orally administered to piglets; LB+ETEC, a basal diet with 0.05% berberine, ETEC orally administered to piglets; HB+ETEC, a basal diet with 0.1% berberine, ETEC orally administered to piglets.

The KEGG pathways that were significantly altered between each two groups were also investigated. A total of 4,139 KEGG pathways were identified as being significantly enriched between the BD+Saline and BD+ETEC groups. Nine KEGG pathways showed significant upregulation (*P*<0.05, log2FC ≥ 1), while 14 exhibited significant downregulation (*P*<0.05, log2FC ≤ −1) (**Figure 9A**). Among these KEGG pathways, 7 (e.g., the Hedgehog signaling pathway and Polycyclic Aromatic Hydrocarbon degradation pathway) were significantly upregulated in the BD+ETEC group. In contrast, 27 KEGG pathways, such as the p53, MAPK, and Hippo signaling pathways, were significantly downregulated in the BD+Saline group (**Figure 9D**). A total of 2,795 KEGG pathways were identified as being altered between the BD+ETEC and LB+ETEC groups. Among these KEGG pathways, 132 showed significant upregulation, while 87 exhibited significant downregulation (**Figure 9B**). Specifically, 20 KEGG pathways, including protein digestion and absorption, insect hormone biosynthesis, and apoptosis, were significantly upregulated in the LB+ETEC group. In contrast, 26 KEGG pathways, including lipoarabinomannan biosynthesis, carotenoid biosynthesis, and the cAMP signaling pathway, were found to be significantly downregulated in the BD+ETEC group (**Figure 9E**). When comparing the BD+ETEC and HB+ETEC groups, a total of 2,712 KEGG pathways were identified as being significantly altered. Among these KEGG pathways, 29 showed significant upregulation, while 52 displayed significant downregulation (**Figure 9C**). Notably, 10 KEGG pathways, such as apoptosis, cholesterol metabolism, and primary bile acid biosynthesis, were found to be significantly upregulated in the HB+ETEC group. Conversely, 37 KEGG pathways, including the cAMP signaling pathway, protein digestion and absorption, and apoptosis, were significantly downregulated in the BD+ETEC group (**Figure 9F**).

### 3.9 Correlation analysis

**Figure 10:**
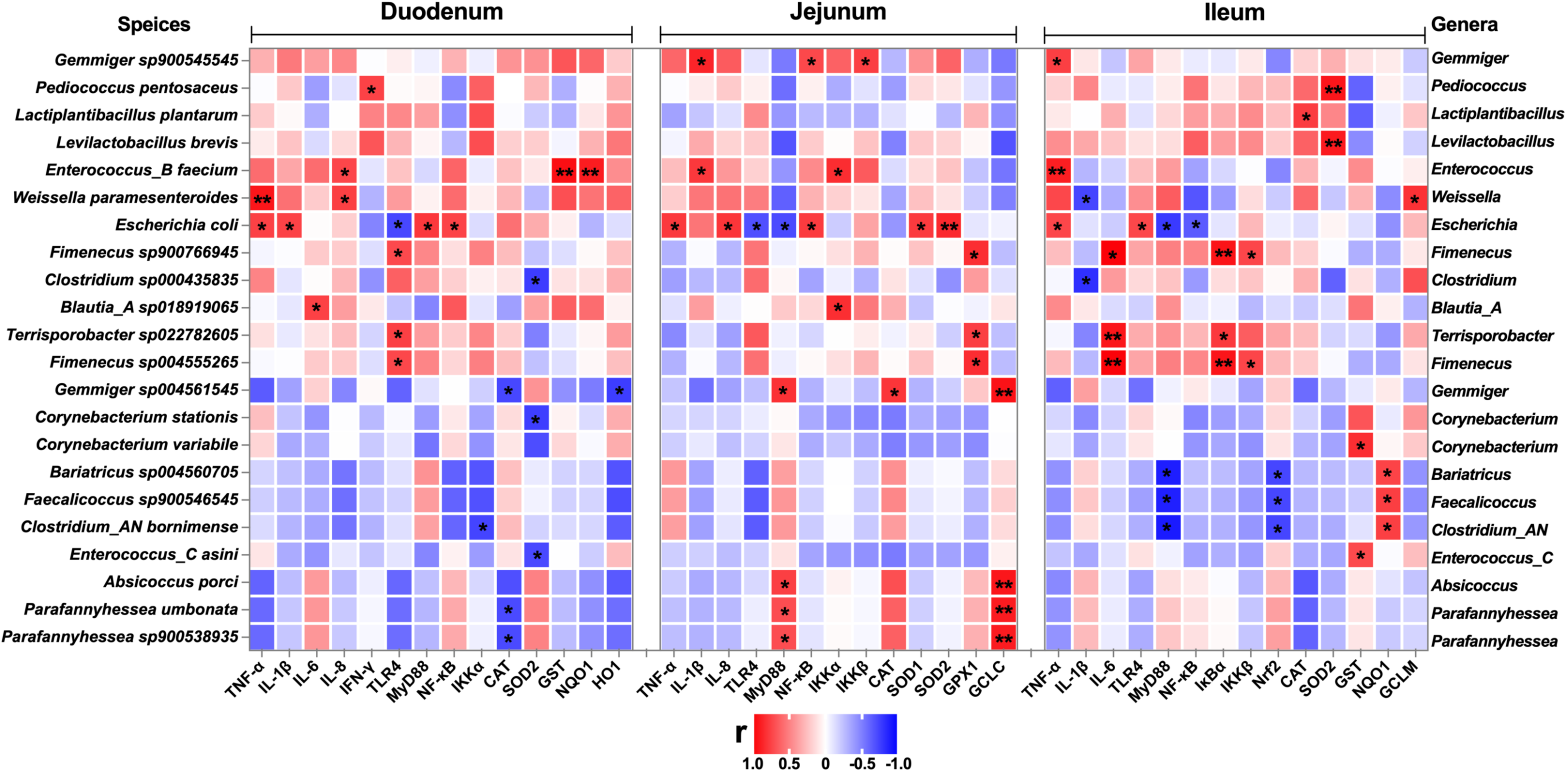
Correlation analysis between the relative abundance of microbiota (BD+Saline *vs*. BD+ETEC, species level) and the expression of inflammation-related genes, antioxidant-related genes, and detoxifying enzyme-related genes in the duodenum, jejunum, and ileum. Red indicates positive correlations and blue indicates negative correlations. ^*^ *P*<0.05, ^**^ *P*<0.0l. BD+Saline, a basal diet with saline orally administered to piglets; BD+ETEC, a basal diet with ETEC orally administered to piglets.

**Figure 11:**
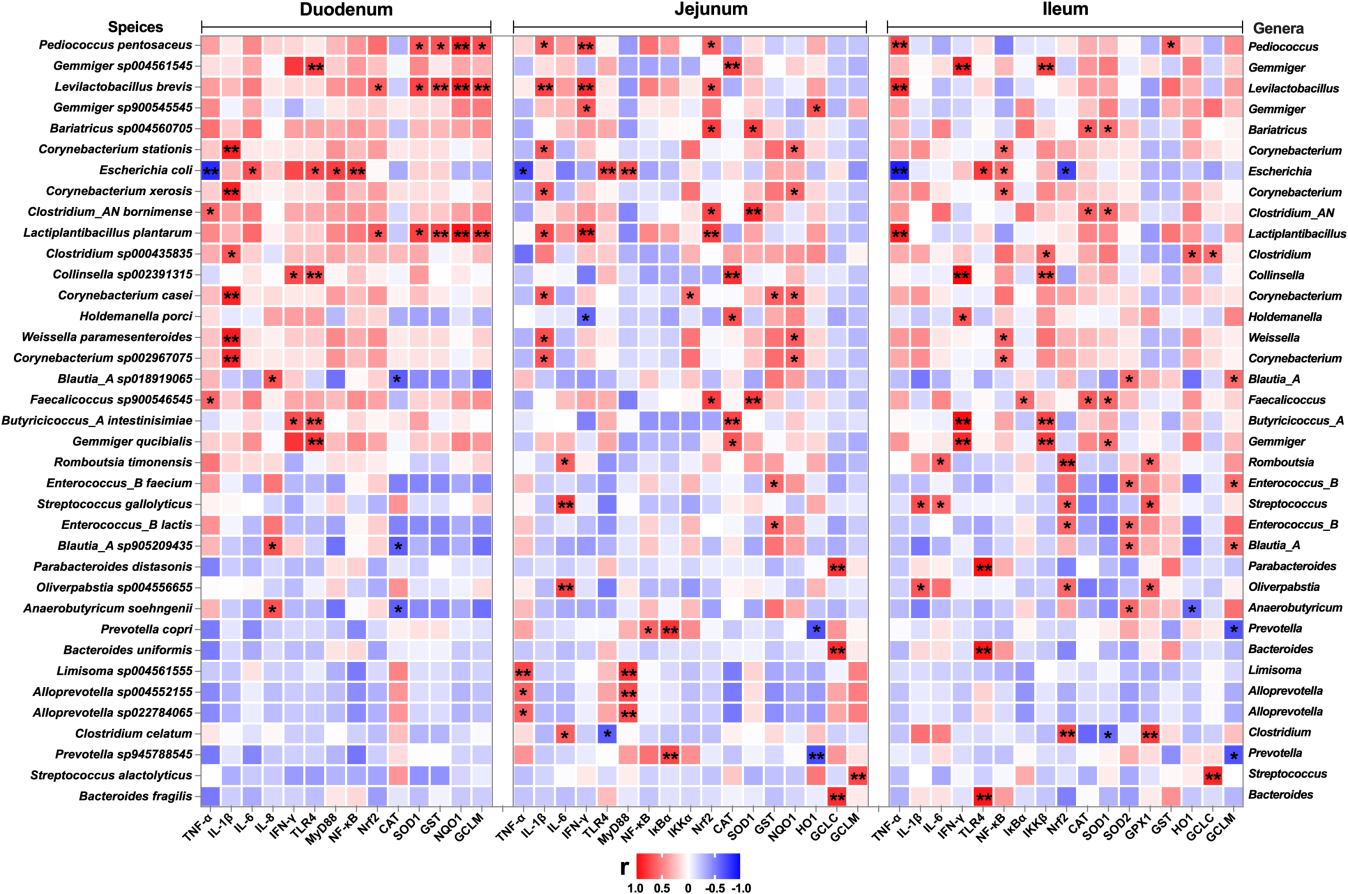
Correlation analysis between the relative abundance of microbiota (BD+ETEC *vs*. LB+ETEC *vs*. HB+ETEC, species level) and the expression of inflammation-related genes, antioxidant-related genes, and detoxifying enzyme-related genes in the duodenum, jejunum, and ileum. Red indicates positive correlations and blue indicates negative correlations. ^*^ *P*<0.05, ^**^ *P*<0.0l. BD+ETEC, a basal diet with ETEC orally administered to piglets; LB+ETEC, a basal diet with 0.05% berberine, ETEC orally administered to piglets; HB+ETEC, a basal diet with 0.1% berberine, ETEC orally administered to piglets.

To investigate the correlation of the intestinal microbiota with the inflammatory response and redox status, we generated a correlation matrix between species-level microbial communities (48 species) and mRNA expression levels (**Figures 10**, **11**) using the Spearman correlation coefficient. A total of 79 significant correlations (58 positive and 21 negative) were determined (**Figure 10**). The relative abundance of *Gemmiger*, *Pediococcus*, *Lactiplantibacillus*, *Levilactobacillus*, *Enterococcus*, *Weissella*, *Escherichia*, *Fimenecus*, *Clostridium*, *Blautia*_A, *Terrisporobacter*, *Corynebacterium*, *Bariatricus*, *Faecalicoccus*, *Absicoccus*, and *Parafannyhessea* was significantly correlated with the inflammatory response (genes encoding TNF-α, IL-1β, IL-6, IL-8, IFN-γ, TLR4, MyD88, NF-κB, IκBα, IKKα, and IKKβ) and the Nrf2 and antioxidant pathway genes (Nrf2, CAT, SOD1, SOD2, GPX1, GST, NQO1, HO1, GCLC, and GCLM).

A total of 159 significant correlations (145 positive and 14 negative) were determined (**Figure 11**). The relative abundance of *Pediococcus*, *Gemmiger*, *Levilactobacillus*, *Bariatricus*, *Corynebacterium*, *Escherichia*, *Lactiplantibacillus*, *Clostridium*, *Collinsella*, *Holdemanella*, *Weissella*, *Blautia*_A, *Faecalicoccus*, *Butyricicoccus*_A, *Romboutsia*, *Enterococcus*, *Streptococcus*, *Parabacteroides*, *Oliverpabstia*, *Anaerobutyricum*, *Prevotella*, *Bacteroides*, *Limisoma*, and *Alloprevotella* was significantly correlated with the inflammatory response (genes encoding TNF-α, IL-1β, IL-6, IL-8, IFN-γ, TLR4, MyD88, NF-κB, IκBα, IKKα, and IKKβ) and the Nrf2 and antioxidant pathway genes (Nrf2, CAT, SOD1, SOD2, GPX1, GST, NQO1, HO1, GCLC, and GCLM).

## 4 Discussion

ETEC is a ubiquitous pathogen in the intestinal microbiota and is known to cause diarrhea in children and piglets, particularly during the neonatal and weaning periods (8). The release of adhesin and enterotoxin by these bacteria in the intestine disrupts the gut microbiota and triggers inflammatory stress, ultimately leading to diarrhea (6, 8, 26). We have previously shown that berberine can improve the composition and structure of the intestinal microbiota of weaned piglets, demonstrate significant antibacterial activity against *Escherichia coli in vitro* (22, 25). In this study, we explored the effects of berberine on the growth performance, intestinal inflammation, and oxidative stress-induced damage of weaned piglets orally challenged with ETEC.

Diarrhea due to ETEC infection has been frequently reported to seriously affect the growth performance of weaned piglets (27). In our study, ETEC infection did not affect the final BW, ADG, ADFI, or FCR of weaned piglets. This result is inconsistent with studies reporting that ETEC infection reduced ADG and ADFI in piglets (27–29). One reason may be that, in our study, we orally challenged the piglets with ETEC for 3 consecutive days following the feeding period, and this short duration of treatment may not have had a significant effect on the growth performance of the weaned piglets. The final BW of the HB+ETEC group, the ADFI of the LB+ETEC group, and the ADG of both the LB+ETEC and HB+ETEC groups were significantly higher than the corresponding parameters in the BD+ETEC group. These results suggested that berberine significantly improves the growth performance of weaned piglets. In addition, ETEC infection can disrupt the intestinal microbiota and intestinal barrier function, leading to the induction of diarrhea (2, 6, 10, 30, 31). Consistent with previous reports (6, 26, 32), we found that the incidence of diarrhea was significantly higher in the BD+ETEC group than in the BD+Saline group, but significantly lower in both the LB+ETEC and HB+ETEC groups. This suggests that dietary berberine can effectively prevent diarrhea in weaned piglets resulting from ETEC infection.

Growing evidence supports that ETEC infection is one of the leading bacterial causes of intestinal inflammation (6, 33, 34). TNF-α, IL-1β, and IL-6 are considered to be biomarkers of inflammation (35). In the present study, ETEC infection led to a significant increase in TNF-α, IL-1β, and IL-6 contents in duodenal, jejunal, and ileal tissues in weaned piglets, effects that were reversed by dietary berberine. These findings align with previous research (36, 37) in which it was shown that ETEC infection induces intestinal inflammation in piglets, while berberine can alleviate such inflammation. Meanwhile, we also found that ETEC infection significantly increased the contents of the anti-inflammatory cytokines TGF‐β and IL‐10 (29, 38) in the duodenum, jejunum, and ileum, while berberine had no significant effect on the contents of these cytokines. During the 3-day ETEC infection process, the inflammatory response induced in the initial stage of infection stimulated the cells of the small intestine of the piglets to produce more TGF‐β and IL‐10, thereby inhibiting inflammation and promoting intestinal tissue recovery from injury (38, 39). The increase in TGF‐β and IL‐10 contents in intestinal tissue after ETEC infection is a protective mechanism against infection and the subsequent inflammatory response (10, 34). Quantifying the expression of genes encoding inflammatory cytokines is crucial for understanding the immune response and the pathological mechanisms involved in infections, such as with ETEC (28, 36). Studies have reported that ETEC infection increases the expression of the genes encoding TNF-α, IL-1β, IL-6, and IL-8 in the small intestine of weaned piglets (28, 40). Consistent with previous studies, our results showed that ETEC infection significantly increased the expression of genes coding for TNF-α, IL-1β, IL-6, and IL-8 in the small intestine of weaned piglets, while berberine supplementation significantly reversed these effects. These results suggest that berberine can alleviate ETEC infection-induced intestinal inflammation in piglets by reducing the levels of TNF-α, IL-1β, IL-6, and IL-8 in the small intestine.

To further elucidate the potential mechanism underlying how berberine mitigates the inflammatory response triggered by ETEC infection, we investigated the TLR4/MyD88/NF-κB signaling pathway. TLR4 serves as the main pathogen recognition receptor for lipopolysaccharides (LPS) derived from Gram-negative bacteria, including ETEC (6, 41). The transcription factor NF-κB plays an integral role in the regulation of inflammatory responses. The activation of NF-κB is mediated by TLR4 through its Toll/Interleukin-1 Receptor (Toll/IL-1R) domain, with MyD88 serving as an adaptor protein. This pathway regulates the expression of genes coding for inflammatory cytokines (42, 43). In our study, ETEC infection significantly increased the expression of TLR4, MyD88, and NF-κB in the small intestine of piglets, while berberine supplementation significantly decreased the expression of these genes. These results suggest that ETEC infection activates the TLR4/MyD88/NF-κB signaling pathway in the intestine, leading to the secretion of a variety of inflammatory factors and triggering intestinal inflammation (6, 41). Furthermore, ETEC infection significantly reduced the expression levels of the IκBα gene and markedly increased those of the genes coding for IKKα and IKKβ in the duodenum and ileum. In contrast, dietary berberine significantly reduced the expression of the latter two genes in both tissues. Studies have shown that IκBα can inhibit NF-κB, maintaining it in an inactive state in the cytoplasm. A complex comprising IKKα/IKKβ triggers the degradation of IκBα through phosphorylation, thereby releasing and activating NF-κB (44, 45). In this study, berberine significantly downregulated the expression of the genes encoding IKKα and IKKβ, consequently reducing the level of IκBα phosphorylation and inhibiting NF-κB activity. This resulted in the modulation of the TLR4/MyD88/NF-κB signaling pathway, leading to the alleviation of the intestinal inflammatory response in piglets caused by ETEC infection.

Oxidative stress and inflammation are highly correlated (27). ETEC infection causes oxidative stress in the small intestine of piglets (7). 8-OHdG is the most frequently detected and studied oxidized DNA product (7) while MDA is the end product of lipid peroxidation and an indicator of oxidative stress (46, 47). In our study, ETEC infection significantly increased the contents of 8-OHdG and MDA in the duodenum and jejunum of piglets, while dietary berberine significantly reversed these increases. These observations implied that ETEC infection caused intestinal oxidative damage in piglets and dietary berberine alleviated oxidative damage by reducing the contents of 8-OHdG and MDA. The T-AOC is an important index for evaluating the overall function of the antioxidant system (46). Our results showed that ETEC infection significantly reduced the T-AOC in the duodenum and jejunum, while dietary berberine had no significant effect on this parameter in the small intestine of piglets. Our results are inconsistent with a previous study that reported that serum T-AOC increased significantly after ETEC infection (48). Based on the results relating to intestinal inflammation and the contents of 8-OHdG and MDA in the intestine, this discrepancy may have arisen because ETEC infection results in the production of large amounts of free radicals and reactive oxygen species (ROS) in the small intestine of piglets, and a substantial amount of antioxidant substances will be consumed when dealing with these harmful substances, resulting in a reduction in the T-AOC.

The initial defense mechanism consists of antioxidant enzymes, including T-SOD, GSH, GSH-Px, and CAT, which serve as the primary antioxidants (27). SOD catalyzes the conversion of superoxide radicals into H_2_O_2_ and O_2_ and is the initial enzyme involved in combating oxyradicals (27). GSH acts as a primary defense mechanism against tissue damage by releasing chemicals that scavenge ROS, enhance cell viability, and stabilize membranes (27, 42). CAT, a major antioxidant enzyme, contributes to alleviating oxidative stress by decomposing cellular hydrogen peroxide, highlighting its importance in various physiological processes (8). Our results showed that ETEC infection significantly increased T-SOD and CAT activities in the duodenum, jejunum, and ileum of piglets, while dietary berberine significantly decreased the contents of these antioxidants in the ileum and jejunum of the animals. Our results are consistent with previous reports (8, 42). This indicates that in the early stage of ETEC infection, to protect against oxidative damage, T-SOD and CAT activities in the small intestine are temporarily increased, while the long-term feeding of berberine in piglets contributes to maintaining the oxidative balance of the small intestine under ETEC infection, explaining why T-SOD and CAT contents tended to decrease with berberine supplementation. We also found that ETEC infection significantly decreased the activities of GSH and GSH-Px in the ileum, whereas the opposite was seen with dietary berberine. This is inconsistent with previous studies, in which it was reported that ETEC infection increased the GSH activity in the serum of piglets (26, 49). One reason for this discrepancy may be that ETEC infection results in extensive ROS production in the small intestine of piglets, leading to the consumption of GSH and GSH-Px in the cells of the small intestine. Through its antioxidant activity, berberine may help restore the activities of GSH and GSH-Px in the small intestine and alleviate the oxidative damage caused by ETEC infection.

Because our results indicated that ETEC infection triggered oxidative stress in the small intestine of weaned piglets, we further examined the Nrf2 signaling pathway, which is a defense mechanism activated by specific stimuli, such as oxidative stress (8). ETEC infection significantly increased the expression level of the *Nrf2* gene in the duodenum, jejunum, and ileum of piglets, indicating that ETEC infection activated the Nrf2 signaling pathway, which is consistent with previous studies (7, 42). Nrf2 acts as a key regulator of antioxidant enzymes and the Nrf2 signaling pathway is crucial for cellular protection against oxidative stress (42). Nrf2 regulates the gene expression of antioxidant factors such as CAT, SOD1, GPX1, and GST, as well as that of phase II detoxifying enzymes, including NQO1, HO-1, GCLC, and GCLM (50). Our study revealed that ETEC infection upregulated the mRNA expression levels of CAT, SOD1, GPX1, GST, NQO1, HO-1, GCLC, and GCLM in the duodenum, jejunum, and ileum of piglets, while dietary berberine significantly downregulated the expression levels of these genes. These results are not entirely consistent with those previously reported, namely, that the expression of these genes was significantly downregulated after ETEC infection in piglets (7, 8, 42, 50, 51). Based on our findings relating to the intestinal inflammatory response and oxidative stress, one possible reason for the above inconsistencies may be that piglets significantly upregulate the expression of antioxidant- and phase (II) detoxifying enzyme-encoding genes in a short time to protect against oxidative damage caused by ETEC infection. Dietary berberine may play a potential antioxidant role and help maintain redox homeostasis in the small intestine of piglets infected with ETEC.

ETEC infection can disrupt intestinal microbial homeostasis, causing an imbalance that leads to detrimental effects and eventually results in diarrhea (6, 10, 52, 53). Accordingly, we undertook a metagenomics analysis of the microbial community in the small intestine of weaned piglets. The results of alpha and beta diversity analysis showed that ETEC infection and berberine altered the diversity of the gut microbiota of the piglets, while Venn diagram and microbial composition analysis demonstrated that ETEC infection and dietary berberine significantly changed the community structure of the intestine of piglets. Dietary berberine altered the composition and structure of the dominant intestinal microbiota, leading to a decrease in microbial abundance. According to the results of our previous research (22, 25), these effects of berberine may lie in the fact that, as a drug and food homology, it may have antibacterial effects similar to antibiotics.

Our results showed that ETEC infection significantly decreased the abundance of *Gemmiger*, *Lactiplantibacillus*, *Enterococcus*, *Levilactobacillus*, *Weissella*, *Clostridium*, *Parabacteroides*, *Phascolarctobacterium*, and *Collinsella*, while dietary berberine increased the abundance of *Gemmiger*, *Pediococcus*, *Levilactobacillus*, *Clostridium*, *Lactiplantibacillus*, *Phascolarctobacterium*, *Weissella*, *Enterococcus*, *Blautia*, and *Butyricicoccus*. Beneficial bacteria, such as *Gemmiger*, play an active role in intestinal inflammation, thereby significantly impacting gut health (54). Similarly, *Lactiplantibacillus* is recognized for its probiotic characteristics and ability to inhibit intestinal inflammatory responses (55). *Enterococcus* spp. are commonly found in the gastrointestinal tract of humans and animals and have been safely used as probiotics (56). Meanwhile, *Levilactobacillus* is known for its potential effect against intestinal pathogens, thereby making a positive contribution to gut health (57). *Weissella* has been identified as a biomarker for healthy microbiota and shows promise as a potential probiotic for promoting intestinal health (58). *Clostridium* comprises a population of volatile fatty acid (VFA)-producing bacteria with anti-inflammatory properties, which contributes to intestinal homeostasis (59, 60). An increased abundance of *Parabacteroides* has been associated with a healthy microbial ecology and reduced inflammation (61, 62). *Phascolarctobacterium* produces VFAs, including acetate and propionate, which are beneficial for gut health (63). In contrast, *Collinsella* is commonly described as a pathobiont linked to compromised intestinal barrier function, indicative of its potential role in gut-related diseases (53, 64). *Pediococcus* is a recognized probiotic with antibacterial activity (65). *Butyricicoccus* and *Blautia* are known to generate short-chain fatty acids (SCFAs) and are thus potentially beneficial (60, 66). Our results also showed that ETEC infection significantly increased the abundance of *Escherichia* and *Streptococcus*; in comparison, dietary berberine decreased the abundance of *Prevotella*, *Streptococcus*, *Parabacteroides*, *Flavonifractor*, and *Alloprevotella*, all of which are reported to be harmful or pathogenic bacteria (61, 67–71). These results indicated that ETEC infection disrupted the intestinal microbiota of weaned piglets. Meanwhile, berberine was found to optimize the gut microbiota in weaned piglets by increasing the abundance of beneficial bacteria and decreasing that of harmful ones.

COG functional annotation of the metagenomic sequencing data showed that ETEC infection significantly upregulated transcription [K], which may be related to the upregulated expression of inflammation- and oxidation-related genes in the small intestine of piglets. In our study, ETEC infection also significantly upregulated inorganic ion transport and metabolism [P], amino acid transport and metabolism [E], and energy production and conversion [C]. ETEC infection typically induces the release of significant amounts of water and electrolytes from intestinal epithelial cells, leading to the primary manifestation of diarrhea (1). Host cells can rebalance electrolytes by increasing the transport of inorganic ions. Under infection, host cells must synthesize a large number of defense-related proteins, and their metabolic activity increases to provide the energy required for cell repair, immune responses, and resistance to pathogens (7, 72). In summary, the upregulation of these functional pathways during ETEC infection represents an adaptive response by host cells to pathogen attack. These reactions help maintain normal cellular functions, promote immune responses, and contribute to the repair of damaged tissues, thereby enhancing resistance to infection. In our study, we found that berberine upregulated the cell wall/membranal/envelope biogenesis [M] and replication, recombination, and repair [L], indicating that berberine promotes the repair and regeneration of the small intestine cell structure of piglets, thereby enhancing the structural integrity and function of the small intestine (73). Berberine significantly enhanced energy production and conversion [C], indicating that it contributes to the provision of energy for the maintenance of the anti-inflammatory and antioxidant functions of the small intestine by upregulating energy metabolism. Dietary berberine also markedly increased intracellular trafficking, secretion, and vesicular transport [U], indicating that berberine can promote the secretion of antimicrobial peptides, cytokines, and other immune-active substances by small intestine cells, which depend on the enhancement of intracellular and vesicular transport mechanisms. Berberine significantly upregulated inorganic ion transport and metabolism [P], showing that berberine helps to restore electrolyte balance, reduce diarrhea and electrolyte disorders, and maintain intracellular acid–base balance, thus ensuring the stability of the intracellular environment (74). In summary, our findings suggested that berberine promotes the repair and regeneration of the cellular structure in the small intestine and provides energy for the maintenance of the anti-inflammatory and antioxidant functions of the small intestine, thereby reducing oxidative stress and inflammation-related damage under ETEC infection. Our data additionally showed that berberine enhances intracellular trafficking, secretion, and vesicular transport, thereby facilitating the secretion of antimicrobial peptides, cytokines, and other immune substances, and promoting the clearance of toxins and metabolic waste. Furthermore, berberine helps to restore electrolyte balance, reduce diarrhea and electrolyte disorders, maintain intracellular acid–base balance, and ensure intracellular environment stability in the small intestine of piglets.

KEGG pathway analysis demonstrated that ETEC infection significantly upregulated non-homologous end-joining and hedgehog signaling pathways. Non-homologous end joining is a mechanism for repairing DNA double-strand breaks (75). In our study, ETEC infection significantly increased the contents of 8-OHdG in the duodenum and jejunum of piglets, suggesting that ETEC infection caused oxidative and DNA damage in the small intestine of the animals (7) and that the enhancement of non-homologous end joining plays a role in maintaining the integrity of cells of the small intestine and protecting them under ETEC infection. ETEC infection significantly upregulates the hedgehog signaling pathway, which participates in cell differentiation, tissue repair, and immune regulation (76). In the initial phases of ETEC infection, the repair of epithelial cells is enhanced in the intestine to counteract the resulting intestinal damage. Our results showed that dietary berberine significantly promoted apoptosis, cholesterol metabolism, protein digestion and absorption, linoleic acid metabolism, glycosphingolipid biosynthesis, primary bile acid biosynthesis, and flavonoid biosynthesis. Dietary berberine plays a multifaceted role in alleviating ETEC infection by modulating distinct metabolic pathways and enhancing the function of cells of the small intestine. Berberine greatly upregulates the apoptotic signaling pathway, aiding in the clearance of ETEC-infected cells and limiting infection spread (77). Moreover, berberine enhances cholesterol metabolism, contributing to the stabilization and strengthening of cell membranes in the small intestine, which, in turn, supports anti-inflammatory and antioxidant signaling, ultimately reducing inflammation and mitigating the damage caused by ETEC (78). Through the upregulation of linoleic acid metabolism, berberine helps in the maintenance and repair of cell membrane integrity, thereby reinforcing the barrier function of intestinal epithelial cells and reducing inflammatory responses (79). Additionally, berberine significantly increases glycosphingolipid biosynthesis, improving the structural and functional capacities of small intestinal cells to combat ETEC infection (80). It also modulates bile acid metabolism, enhancing intestinal antibacterial defenses, and stimulates the biosynthesis of flavonoids with anti-inflammatory, antioxidant, and antibacterial properties, thus aiding in the alleviation of inflammation and oxidative stress (81, 82). These combined effects highlight the therapeutic potential of berberine in managing ETEC infections and improving the gut health of weaned piglets.

To investigate the connection between ETEC infection and intestinal inflammation and oxidative damage in piglets, we examined the relationship between the notable shifts in microbial abundance at the genus level and the expression levels of genes related to intestinal inflammation and oxidative stress between the BD+Saline and BD+ETEC groups. Studies have shown that decreased *Gemmiger* abundance is related to increased levels of inflammation in patients with inflammatory bowel disease (83). *Gemmiger* regulates the development of nonalcoholic fatty liver disease by affecting oxidative stress response (84). Our results are consistent with these reports, indicating that the abundance of *Gemmiger* was significantly correlated with the inflammatory response (IL-1β, MyD88, NF-κB, IKKβ) and oxidative stress (CAT, GCLC) in the jejunum. These findings further implied that the decrease in the relative abundance of *Gemmiger* caused by ETEC infection enhanced intestinal inflammation and oxidative damage in weaned piglets. *Lactiplantibacillus* and *Levilactobacillus* are two genera of lactic acid bacteria known for their beneficial roles in reducing oxidative stress (85). Here, we found a significant correlation between the relative abundance of *Lactiplanitibacillus* and *Levitactobacillus* and the ileal antioxidant capacity (CAT, SOD2). *Enterococcus* was significantly associated with inflammation in the duodenum (IL-8), jejunum (IL-1β and IKKα), and ileum (TNF-α), as well as the antioxidant capacity in the duodenum (SOD2, GST, NQO1) and jejunum (GST), consistent with previous reports (56, 70, 86). *Weissella* is widely recognized as a nonpathogenic and probiotic bacterium that helps reduce intestinal inflammation by inhibiting the production of proinflammatory cytokines (87). *Weissella parameteroides* has significant free radical scavenging ability, which helps alleviate intestinal oxidative stress (58). Consistent with these studies, we noted that the relative abundance of *Weissella* was significantly correlated with inflammation in the duodenum (TNF-α and IL-8) and ileum (IL-1β), as well as with the antioxidant capacity in the ileum (GCLM). *Clostridium* can reduce intestinal inflammation by producing butyric acid (60) and can counteract oxidative stress and protect host cells against oxidative damage through its metabolites (59). Similarly, we found a significant correlation between *Clostridium* abundance and inflammation in the duodenum (IKKα) and ileum (IL-1β), as well as between *Clostridium* abundance and the antioxidant capacity in the duodenum (SOD2) and ileum (Nrf2, NQO1). ETEC infection resulted in a significant increase in the relative abundance of *Escherichia coli*. Additionally, the relative abundance of *Escherichia coli* was significantly correlated with the inflammatory response in the duodenum (TNF-α, IL-1β, TLR4, MyD88, NF-κB), jejunum (TNF-α, IL-8, TLR4, MyD88, NF-κB) and ileum (TNF-α, TLR4, MyD88, NF-κB). Consistent with the previous inflammatory results, ETEC infection is the main inducer of intestinal inflammation in piglets (48). In summary, the alterations in the abundance of various microorganisms in the intestine of piglets under ETEC infection are intricately linked to the intestinal inflammatory response and oxidative stress. Specifically, a decrease in the abundance of beneficial bacteria (*Gemmiger*, *Lactiplantibacillus*, *Enterococcus*, *Levilactobacillus*, *Weissella*, and *Clostridium*) leads to the intensification of the intestinal inflammatory response and oxidative damage, while an increase in the abundance of pathogenic bacteria (*Escherichia coli*) significantly promotes intestinal inflammatory responses.

Our results showed that berberine significantly increased the relative abundance of beneficial bacteria (*Gemmiger*, *Pediococcus*, *Levilactobacillus*, *Clostridium*, *Lactiplantibacillus*, *Weissella*, *Enterococcus*, *Blautia*, and *Butyricicoccus*) and decreased that of harmful ones (*Prevotella*, *Streptococcus*, *Parabacteroides*, *Flavonifractor*, and *Alloprevotella*). We also analyzed the correlation between the relative abundance of these bacteria and the intestinal inflammatory response and oxidative stress marker genes. *Pediococcus pentosaceus*, a probiotic, has been shown to inhibit the production of proinflammatory cytokines (88). It also has antioxidant activity and mitigates oxidative stress-induced damage to cells of the small intestine (89). Consistent with this, our results showed that the relative abundance of *Pediococcus pentosaceus* was significantly correlated with the inflammatory response in the jejunum (IL-1β, IFN-γ) and ileum (TNF-α) and with oxidative stress in the duodenum (SOD1, GST, NQO1, GCLM) and jejunum (Nrf2). *Blautia* modulates the intestinal inflammatory response by regulating gut microbial balance and inhibiting proinflammatory cytokine production (60, 90). Furthermore, bacteria in this genus can enhance the antioxidant capacity of the small intestine and mitigate oxidative stress-induced damage to intestinal cells by producing metabolites such as SCFAs (60). Similarly, in our study, the abundance of *Blautia* was significantly correlated with inflammation in the duodenum (IL-8) and the antioxidant capacity in the duodenum (CAT) and ileum (SOD2, GCLM). *Butyricoccus* plays a crucial role in modulating inflammatory responses and oxidative stress through the production of SCFAs such as butyric acid (66). Our results showed that the abundance of *Butyricoccus* was significantly correlated with the inflammatory response in the duodenum (IFN-γ, TLR4) and ileum (IFN-γ, IKKβ) and with the antioxidant capacity in the jejunum (CAT). In addition, berberine increased the relative abundance of beneficial bacteria (*Gemmiger*, *Levilactobacillus*, *Clostridium*, *Latiplantibacillus*, *Weissella*, *Enterococcus*) and significantly reduced that of pathogenic bacteria (*Prevotella*, *Streptococcus*, *Parabacteroides*, *Flavonifractor*, and *Alloprevotella*), genera that are closely related to intestinal inflammation and oxidative stress in piglets. In summary, dietary berberine increased the abundance of beneficial bacteria and decreased that of harmful bacteria in the intestine of weaned piglets, thereby alleviating the intestinal inflammatory response and oxidative damage caused by ETEC infection.

## 5 Conclusions

In this study, we investigated the impact of berberine on the growth performance, intestinal inflammatory response, oxidative damage, and intestinal microbiota of weaned piglets infected with ETEC. We found that dietary berberine significantly enhances growth performance, optimizes intestinal microecology, and alleviates intestinal inflammation and oxidative damage in weaned piglets challenged with ETEC. Berberine supplementation was found to improve the final BW, ADG, and ADFI, while effectively reducing the incidence of diarrhea in piglets. ETEC infection disrupts the intestinal microbiota, leading to the activation of the TLR4/MyD88/NF-κB signaling pathway, and, consequently, intestinal inflammation in weaned piglets. Additionally, ETEC infection activates the Nrf2 signaling pathway to protect against oxidative damage in the small intestine. Berberine can optimize intestinal microbiota balance by enhancing the abundance of beneficial bacteria, reducing that of harmful bacteria, and modulating the TLR4/MyD88/NF-κB and Nrf2 signaling pathways, thereby helping to alleviate the intestinal inflammation and oxidative damage caused by ETEC infection in piglets. These findings suggest that berberine has the potential for use as a dietary supplement to improve the health and growth of weaned piglets by enhancing their intestinal health and resilience against ETEC challenges.

## Data Availability

The datasets presented in this study can be found in online repositories. The names of the repository/repositories and accession number(s) can be found below: https://www.ncbi.nlm.nih.gov/, SRR 29048499 to 29048514.
