# Supplementary material for: Berberine alleviates ETEC-induced intestinal inflammation and oxidative stress damage by optimizing intestinal microbial composition in a weaned piglet model
Source: Front Immunol. 2024 Sep 16;15:1460127. doi: 10.3389/fimmu.2024.1460127 (PMC11440249; doi:10.3389/fimmu.2024.1460127)
Supplement: Supplementary file 1 [file Table1.docx]

Supplementary Material

Berberine alleviates ETEC-induced intestinal inflammation and oxidative stress damage by optimizing intestinal microbial composition in a weaned piglet model

Yue Wang^1^, Ziting Zhang^1^, Min Du^1^, Xu Ji^3^, Xiaodan Liu^1^, Chunfang Zhao^1,2^, Xunsheng Pang^1^, Erhui Jin^1,2^, Aiyou Wen^1^, Shenghe Li^1,2^, Feng Zhang^1,2*^

^1^College of Animal Science, Anhui Science and Technology University, Chuzhou, China

^2^Anhui Province Key Laboratory of Animal Nutrition Regulation and Health, Chuzhou, China

^3^Anhui Province Key Laboratory of Livestock and Poultry Product Safety Engineering, Institute of Animal Science and Veterinary Medicine, Anhui Academy of Agricultural Sciences, Hefei, China

*** Correspondence:**Feng Zhang
zhangfeng@ahstu.edu.cn

# Supplementary Tables

**Table S1.** Ingredients composition and nutrient specification of basal diet

| Items (%) | Basal diet |
| --- | --- |
| Corn | 37.50 |
| Extruded corn | 15.00 |
| Soybean meal | 15.00 |
| Extruded soybean | 10.00 |
| Whey power | 10.00 |
| Vitamin and mineral premix* | 4.00 |
| Fish meal | 2.50 |
| Egg yolk powder | 2.00 |
| Sucrose | 2.00 |
| Soybean oil | 2.00 |
| Analyzed nutrient specification (%) | |
| Dry matter | 88.2 |
| Crude ash | 5.80 |
| Crude protein | 17.04 |
| Ether extract | 5.60 |
| Crude fiber | 2.80 |
| Ca | 0.54 |
| Total P | 0.54 |

*Provided per kilogram of diet: Zn (ZnSO_4_·H_2_O), 100 mg; Cu (CuSO_4_·5H_2_O), 125 mg; Mn (MnSO_4_·H_2_O), 60 mg; Fe (FeSO_4_·H_2_O), 120 mg; I (Ca(IO_3_)_2_, 0.6 mg; Se (Na_2_SeO_3_), 0.30 mg; vitamin A, 10000 IU; vitamin D3, 2500 IU, vitamin 35 IU; vitamin K3, 3.0 IU; Vitamin B5, 40 mg; nicotinic acid, 60 mg; folic acid, 1 mg; biotin, 0.2 mg; vitamin B6, 4.0 mg; vitamin B2, 7.5 mg; vitamin B1, 5.0 mg; vitamin B12, 0.08 mg.

**Table S2.** Sequences of genes-special primers used for RT-qPCR.

| Genes | Accession number | Primer sequences (5’-3’) |
| --- | --- | --- |
| TNF-α | NM_214022.1 | F: TTCTGCCTACTGCACTTCGAG  R: AGGGCATTGGCATACCCAC |
| IL-1β | NM_214055.1 | F: CCAGCCAGTCTTCATTGTTCAG  R: GCTGGATGCTCCCATTTCTC |
| IL-6 | NM_214399.1 | F: TCCTCGGCAAAATCTCTGCAA  R: ACAAGACCGGTGGTGATTCTC |
| IL-8 | NM_213867.1 | F: GCCTTCTTGGCAGTTTTCCTG  R: TGGAAAGGTGTGGAATGCGTA |
| IFN-γ | NM_213948.1 | F: CAGCTTTGCGTGACTTTGTG  R: GGTCCACCATTAGGTACATCTGA |
| TLR4 | NM_001113039.2 | F: CTCCAGCTTTCCAGAACTGC  R: AGGTTTGTCTCAACGGCAAC |
| MyD88 | NM_001099923.1 | F: TGGAACAGACCAACTATCGGC  R: CATCAGAGACAACCACTACCATCC |
| NF-κB | NM_001114281.1 | F: GCTACACAGGACCAGGAACAGTTC  R: CTTGCTCCAGGTCTCGCTTCTTC |
| IκBα | NM_001005150.1 | F: CAGAATCCCGACCTGGTGTC  R: TGAGCTGGTACGGGGAGTAG |
| IKKα | NM_001114279.1 | F: TCTGGAACAGCGTGCCATTGATC  R: TCCTTGAGAACACGGTCCTGACTC |
| IKKβ | XM_021077171.1 | F: GTGACATCGCCTCTGCACTTA  R: CTTGGCATACCCGAGGTCAA |
| Nrf2 | XM_021075133.1 | F: CCCATTCACAAAAGACAAACATTC  R: GCTTTTGCCCTTAGCTCATCTC |
| CAT | NM_214301.2 | F: TCCAGCCAGTGACCAGATGA  R: CTCTCCCGGTCAAAGTGAGC |
| SOD1 | NM_001190422.1 | F: AAGGCCGTGTGTGTGCTGAA  R: GATCACCTTCAGCCAGTCCTTT |
| SOD2 | NM_214399.1 | F: GGCCTACGTGAACAACCTGA  R: TGATTGATGTGGCCTCCACC |
| GPX1 | NM_214201.1 | F: TGAATGGCGCAAATGCTCAC  R: ATTGCGACACACTGGAGACC |
| GST | NM_214389.2 | F: CAGCTGCAACAACAGCTTACAA  R: AGCCACCGGATACACTCCAT |
| NQO1 | NM_001159613.1 | F: GCCCAGATATTGTGGCCGAA  R: AACTCCCCTATGAGCACACG |
| HO1 | NM_001004027.1 | F: TCAAGCAGAGGGTCCTCGAA  R: CCTCTTGCGGATGTCGGATG |
| GCLC | XM_021098556.1 | F: GACGACGCCAATGAGTCTGA  R: AGCACCACAAACACCACGTA |
| GCLM | XM_001926378.4 | F: GATGCCGCCCGATTTAACTG  R: ACAATGACCGAGTACCGCAG |
| β-actin | XM_021086047.1 | F: GGACTTCGAGCAGGAGATGG  R: GCACCGTGTTGGCGTAGAGG |
| GAPDH | NM_001206359.1 | F: TCGGAGTGAACGGATTTGGC  R: CACCCCATTTGATGTTGGCG |
